# Genome-Wide Identification and Expression Analysis of SlNRAMP Genes in Tomato under Nutrient Deficiency and Cadmium Stress during Arbuscular Mycorrhizal Symbiosis

**DOI:** 10.3390/ijms25158269

**Published:** 2024-07-29

**Authors:** Junli Liu, Xiaoqi Bao, Gaoyang Qiu, Hua Li, Yuan Wang, Xiaodong Chen, Qinglin Fu, Bin Guo

**Affiliations:** 1 State Key Laboratory for Managing Biotic and Chemical Threats to the Quality and Safety of Agro-Products, Institute of Environment, Resource, Soil and Fertilizer, Zhejiang Academy of Agricultural Sciences, Hangzhou 310021, China; liujunli@zaas.ac.cn (J.L.); 211122270076@zjut.edu.cn (X.B.); qiugy@zaas.ac.cn (G.Q.); lihua@zaas.ac.cn (H.L.); wangyuan@zaas.ac.cn (Y.W.); chenxiaodong@zaas.ac.cn (X.C.); fuql@zaas.ac.cn (Q.F.); 2 College of Environment, Zhejiang University of Technology, Hangzhou 310032, China

**Keywords:** arbuscular mycorrhizal fungi, cadmium, gene expression, NRAMP, tomato

## Abstract

Arbuscular mycorrhizal (AM) fungi are well known for enhancing phosphorus uptake in plants; however, their regulating roles in cation transporting gene family, such as natural resistance-associated macrophage protein (NRAMP), are still limited. Here, we performed bioinformatics analysis and quantitative expression assays of tomato *SlNRAMP* 1 to 5 genes under nutrient deficiency and cadmium (Cd) stress in response to AM symbiosis. These five SlNRAMP members are mainly located in the plasma or vacuolar membrane and can be divided into two subfamilies. Cis-element analysis revealed several motifs involved in phytohormonal and abiotic regulation in their promoters. *SlNRAMP2* was downregulated by iron deficiency, while *SlNRAMP1*, *SlNRAMP3*, *SlNRAMP4,* and *SlNRAMP5* responded positively to copper-, zinc-, and manganese-deficient conditions. AM colonization reduced Cd accumulation and expression of *SlNRAMP3* but enhanced *SlNRAMP1*, *SlNRAMP2,* and *SlNRMAP4* in plants under Cd stress. These findings provide valuable genetic information for improving tomato resilience to nutrient deficiency and heavy metal stress by developing AM symbiosis.

## 1. Introduction

Natural resistance-associated macrophage proteins (NRAMPs) are among the most important metal transporters. They play a crucial role in transporting and balancing divalent metal cations, such as iron (Fe), manganese (Mn), copper (Cu), and zinc (Zn) in plants [1,2,3]. Previous studies have extensively investigated the functions of NRAMP proteins in various plant species, including rice (*Oryza sativa* L.) [4], duckmeat *(Spirodela polyrhiza)* [5], thale cress (*Arabidopsis thaliana*) [6], wheat (*Triticum aestivum* L.) [7], soybean (*Glycine max* L.) [8], peanut (*Arachis hypogaea* L.) [9], potato (*Solanum tuberosum* L.) [10], black cottonwood (*Populus trichocarpa*) [11], and *Sedum alfredii* Hance [12]. Different members of the NRAMP gene family have been shown to have different functions. For example, in thale cress, *AtNRAMP1* regulates Mn and Fe levels in the roots under Mn shortage [13], while *AtNRAMP3* and *AtNRAMP4* transport both Fe and Mn [6,14]. *AtNNRAMP6* can transport Fe and Cd in yeast cells, and its disruption leads to decreased growth of lateral roots in thale cress plants, especially under Fe deficiency [11,15]. In rice, *OsNRAMP2* plays a role in Fe remobilization during seed germination and Cd accumulation in grains [16], while *OsNRAMP3* facilitates Mn transportation to young leaves under limited Mn availability [17]. *OsNRAMP4* is involved in the movement of Al and Cd accumulation in grains [18], and *OsNRAMP5* is responsible for the uptake of Fe, Mn, Cd, and Pb [18,19].

Genomic modification has further confirmed the transporting role of NRAMPs. For instance, silencing *StNRAMP2* leads to increased Cd accumulation in tubers but decreased accumulation in other tissues, while overexpressing the *StNRAMP2* gene in tomato increases Cd content [20]. Mutants with impaired *ZmNRAMP2* function show a reduced Mn concentration in the xylem sap and retention of Mn in the root stele [21]. *MsNRAMP1* is involved in the process of arbuscular mycorrhizal fungi (AMF) regulating the accumulation of Cd from alfalfa roots to the aboveground parts [22].

Arbuscular mycorrhizal fungi (AMF) are micro-fungi belonging to the phylum Glomeromycota, widely known for their symbiotic association with roots in over 90% of land plants [23,24,25,26]. Once the symbiotic association is established, the AMF receive up to 20% of the plant-fixed carbohydrates from their host plants, while in return, they improve the supply of important mineral nutrients, particularly phosphorus [26,27]. Additionally, Arbuscular mycorrhizal fungi can affect the absorption, accumulation, and detoxification of heavy metals in plants via the direct uptake by their hyphae [28,29,30,31,32]. Furthermore, Arbuscular mycorrhizal fungi can regulate DNA methylation in host cells and modulate the expression of genes associated with metal tolerance and detoxification [24,33]. For instance, Arbuscular mycorrhizal fungi decrease the uptake of Cd in rice by altering the expression of *OsNRAMP5* and *OsHMA3* [34]. Similar results have been found in alfalfa (*Medicago sativa* L.) [22], soybean [35], and pea (*Pisum Sativum* L.) [36]. However, the mechanism underlying NRAMP regulation by AMF is still largely unknown.

Tomato is a widely consumed and versatile fruit that holds significant importance in various aspects of human life. It is also a valuable model plant for biological and genetic research due to its relatively low-copy DNA sequence and its nearly complete genome sequencing [37,38,39]. Mineral nutrients are indispensable for the normal growth and development of cucurbit crops, such as micronutrients Fe, Mn, Cu, and Zn, but nutrient deficiency in the natural environment can have a detrimental impact on the growth and quality of tomatoes [40,41,42]. Furthermore, it is a Cd-sensitive indicator plant [37,43] and can form a suitable symbiotic relationship with AMF [26,44,45]. Although *SlNRAMP1* has been confirmed to be involved in Fe mobilization in the vascular parenchyma upon Fe deficiency in plants [46], limited information regarding the potential interactions between AM symbiosis and NRAMP-mediated Cd uptake is available so far. In this study, we performed bioinformatics analysis and quantitative expression assays of five tomato SlNRAMP genes, SlNRAMP1 to 5, under nutrient deficiency and Cd stress in response to AM symbiosis. These findings herein provide valuable information for further studies on the physiological roles of SlNRAMPs in the uptake of Cd and mineral nutrients during AM symbiosis.

## 2. Results

### 2.1. Identification and Phylogenetic Analysis of the SlNRAMP Family Genes in Tomato

The coding sequences (CDSs) of the three accessioned tomato NRAMP genes and homolog from the thale cress NRAMP family (AtNRAMP1–6) and rice NRAMP family (OsNRAMP1–7) were used as query sequences. Following BLAST searches of the tomato genomic sequence database, seven potential SlNRAMP genes were identified. However, two sequences were excluded as they only had zero and three transmembrane structures. Ultimately, five distinct genomic sequences encoding putative isoforms of SlNRAMPs were identified, including the previously accessioned tomato SlNRAMP members, which were renamed SlNRAMP1–5. The full details can be found in Table 1 and Figure S1.

Table 1 illustrates the gene sequence analyses of five SlNRAMP genes. The shortest gene length (1853 bp) belongs to tomato SlNRAMP1; however, both the smallest coding sequence (1347 bp) and amino acid sequence (509) are found in tomato SlNRMAP3. Moreover, the largest gene length (4841 bp), coding sequence (3951 bp), and number of amino acids (1316) are found in tomato SlNRMAP5. Furthermore, the SlNRAMP proteins exhibited different physicochemical properties, including PI (isoelectric points), instability, aliphatic index, and GRAVY. The PI ranged from 5.23 (SlNRAMP2) to 8.85 (SlNRAMP4), whereas instability ranged from 29.73 (SlNRAMP3) to 40.57 (SlNRAMP5). Additionally, the aliphatic index and the GRAVY were both smallest in tomato SlNRAMP5 (88.76 and −0.013), but they are both largest in tomato SlNRAMP1 (124.55 and 0.6). Moreover, the minimum molecular weight was 56.15 kDa (SlNRAMP3), while the maximum molecular weight was 142.67 kDa (SlNRAMP5). All SlNRAMP proteins, except SlNRAMP2, which had 9 transmembrane regions, contained more than 10 transmembrane regions. The SlNRAMP family exhibited different localizations, with SlNRAMP2 and SlNRAMP3 predicted to be localized in the vacuole membrane, SlNRAMP1 and SlNRAMP5 in the plasma membrane, and SlNRAMP4 possibly in the vacuole membrane and plasma membrane.

**Figure 1:**
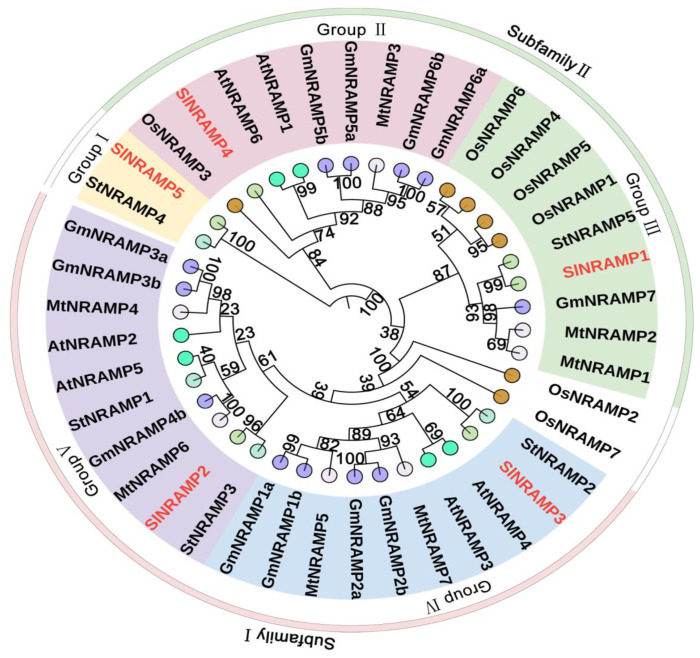
Phylogenetic analysis of SlNRAMP family proteins and the other homologs.

To understand the functions and evolutionary relationships of tomato NRAMP genes with other plant homologs, a maximum likelihood tree was constructed using 42 NRAMP protein sequences from model plants (rice and thale cress), solanaceous plants (potato and tomato (*Solanum lycopersicum*)), and two leguminous plants (soybean and alfalfa). The resulting analysis revealed that NRAMPs were divided into two major subfamilies (Figure 1 and Table S1). The basal cluster consisted of StNRAMP4 and SlNRAMP5, indicating that a single ancestral NRAMP gene underwent independent duplications in the solanaceous lineages after the split of Solanaceae. Subfamily I (Groups VI–V), represented by AtNRAMP2/3/4/5, included two members of the SlNRAMP family. Subfamily II (Groups II–III), characterized by AtNRAMP1/6 and OsNRAMP1/3/4/5/6, comprised two members of the SlNRAMP family (Figure 1). Interestingly, most dicotyledonous plant species, such as potato, tomato, and soybean, were located on adjacent branches, indicating strong genetic relationships.

The unrooted phylogenetic tree was constructed using the maximum likelihood method within the MEGA 6 program. The species involved in the evolutionary tree include tomato (SlNRAMP1–5), thale cress (AtNRAMP1–6), rice (OsNRAMP1–7), alfalfa (MtNRAMP1–7), potato (StNRAMP1–5), and soybean (GmNRAMP1a–3a,5a–6a,7,1b–6b).

### 2.2. Domains, Conserved Motifs, and Models of the SlNRAMP Family Proteins

**Figure 2:**
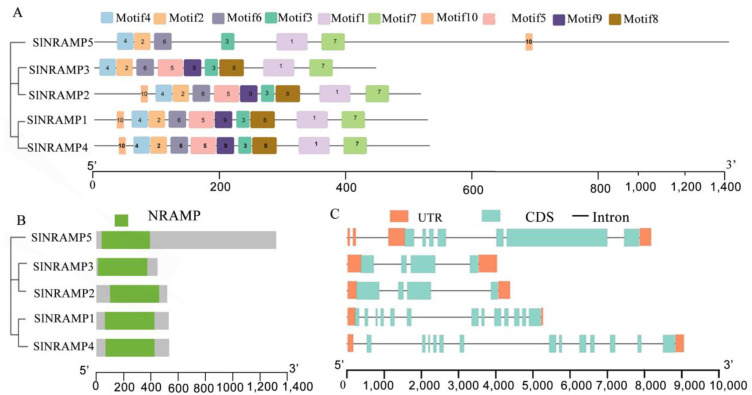
Conserved motifs and exon/intron structures of the five SlNRAMP genes. Conserved motifs (**A**) and domains (**B**) in SlNRAMP family proteins as well as exon–intron structure (**C**) of NRAMP genes from tomato. UTR and CDS represent untranslated regions and coding sequences, respectively.

Using MEME, we identified 10 conserved motifs in the SlNRAMP family protein sequences (Table S2 and Figure 2A). These motifs were 29–50 amino acids in length and closely related to motifs and structures. The SlNRAMP family proteins all contained a unique NRAMP domain within the NRAMP metal transporter family, which had the consensus residue IWAIGLLAAGQSSTITGTYAGQFIMGGFLDLRLKKWLRALI-TRSCAIVP (Table S2). All SlNRAMP family proteins, except for SlNRAMP5, contained nine of the identified motifs (Figure 2A). The motif distribution and quantity were similar among genes in the same branch of the evolutionary tree. For instance, SlNRAMP1 and SlNRAMP4 shared the same motif compositions (motifs 1–10), while SlNRAMP2 and SlNRAMP3 shared nine of the same motifs, except for motif 10. SlNRAMP5 had the lowest number of motifs (Figure 2A). The NRAMP domain was present in all SlNRAMP proteins according to the Pfam tool (Figure 2B), suggesting that different motif distributions in different groups may have led to functional diversity during the evolutionary process. The conserved motifs of NRAMP genes within the same group implied similar functions.

Based on Figure 2C, the tomato NRAMP family could be divided into three subfamilies, each displaying unique variations in exon and intron quantity and distribution when compared to both mRNA and genomic sequences. Group I, represented by SlNRAMP5, contained seven exons and six introns, while both SlNRAMP2 and SlNRAMP3 in Group II demonstrated fewer exons and introns. In contrast, SlNRAMP1 and SlNRAMP4 in Group III showed a highly fragmented gene structure, with 13 and 12 introns, respectively. The gene structure differed significantly between the phylogenetic groups but remained highly conserved within each group. These results provide evidence of distinct subfamilies within the tomato NRAMP family, each with diverse gene structures.

### 2.3. Chromosomal Organization and Duplication of the SlNRAMP Family Genes

**Figure 3:**
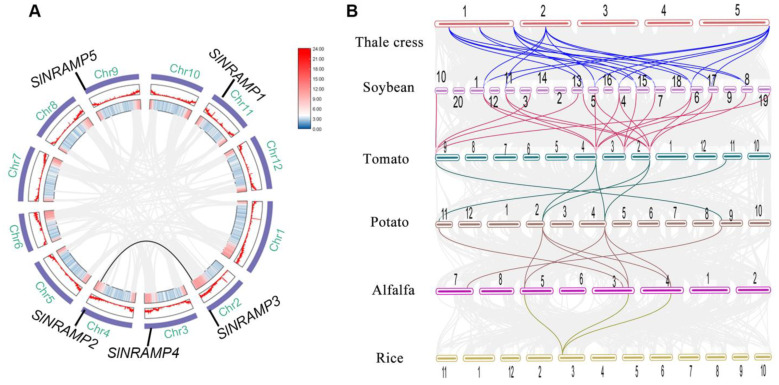
Chromosomal location and synteny relationship of SlNRAMP family genes pairs in tomato and other five species. Chromosomal location and synteny relationship of SlNRAMP gene pairs in tomato (**A**); synteny relationship of NRAMP gene pairs among five plant species (**B**). The color lines represent synteny genes. The gray lines show the collinear blocks of the plant genomes.

The distribution of the five tomato SlNRAMP members was uneven across five tomato chromosomes (2, 3, 4, 9, and 11), as illustrated in Figure 3A and Table S3. Furthermore, collinearity analysis of SlNRAMP family genes in tomato revealed only one pair of genes, SlNRAMP2 on chromosome 4 and SlNRAMP3 on chromosome 2, indicating their high homology (Figure 3A).

To provide evidence of the evolutionary process in tomato, we conducted a thorough examination of the syntenic relationships among NRAMP gene pairs in various species. A collinear map of the NRAMP family genes was generated for six plant species, comprising model plants (rice and thale cress), solanaceous plants (potato and tomato), and two leguminous plants (soybean and alfalfa). In total, 51 colinear gene pairs were identified, with significant numbers of collinear gene pairs detected between thale cress and soybean (17 collinear gene pairs, Figure 3B and Table S4), as well as between soybean and tomato (20 collinear gene pairs, Figure 3B and Table S4), indicating genetic duplication during the evolution of these species. Notably, several uninterrupted colinear gene pairs were observed across various species, indicating that these homologous genes were established before species divergence.

### 2.4. Multiple Sequence Alignment and 3D Model Predictions of the SlNRAMP Family Proteins

**Figure 4:**
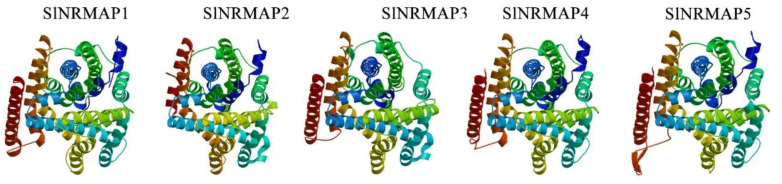
Predicted 3D structure of the five SlNRAMP family proteins by SwissModel. Models were visualized by rainbow color from N to C termini.

Currently, the crystal structure data for tomato SlNRAMP family proteins are unavailable in the protein database, making their exact structures unclear. The predicted models of tomato NRAMP proteins were visualized by rainbow color from the N to the C termini (Figure 4). Based on our analysis, the template 5m8k.1.A (crystal structure of a divalent metal transporter from Eremococcus coleocola EcoDMT (PDB ID 5m8k)) was found to be a suitable match for all SlNRAMP proteins (Figure 4 and Table S5). The QMEAN-DisCo global score ranged from 0.59 to 0.65, and the GMQE ranged from 0.58 to 0.66, except for SlNRAMP5 (GMQE: 0.2) (Table S5). Additionally, all sequence identity values were greater than 24.56%. These results suggest that the 3D protein structure predictions for tomato NRAMP proteins are of high quality.

**Figure 5:**
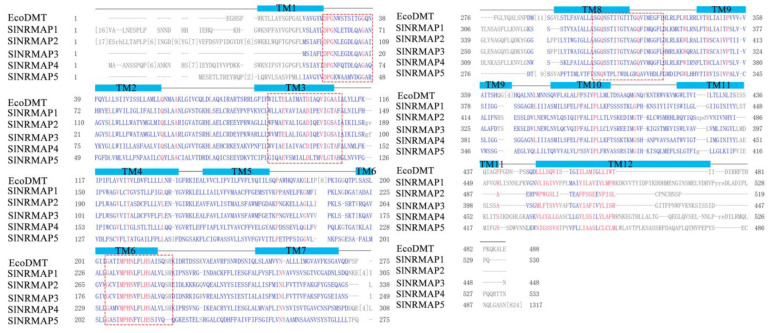
Multiple sequence alignment of SlNRAMP family protein sequences. Multiple sequence alignment columns with no gaps are colored in blue or red. The red color indicates highly conserved columns, and blue indicates less conserved ones. Red is for conserved residues, and blue is for columns with no gaps. Gray is for columns containing gaps. Where less than 50% of the sequences contain gaps, they are shown in gray uppercase. Greater than 50% will be gray lowercase. But the unaligned columns are compressed into the bracket form: [x], where x denotes the number of residues for a sequence in the unaligned range. Red dotted box indicated highly conserved amino acid residues.

For further analysis of the considerable homology and conservation between tomato SlNRAMP family proteins and EcoDMT, protein homology analysis was conducted using a constraint-based multiple-alignment tool. As shown in Figure 5, the tomato NRAMP gene family was highly conserved and variational compared with EcoDMT containing 12 transmembrane domains (TMDs). GQSSTITGTYAGQY(/F)V(/I)MQ(/E)GFL(/I), located in TM8, TM9, and the region between them, was highly conserved in SlNRAMP1–4 and EcoDMT but was partially variated in the SlNRAMP5 protein sequence. Moreover, certain highly conserved amino acid residues existed mainly in TMDs, such as DPGN(K) in TM1, DI(/L)Q(/P/T/A)E(/Q/M)VI(/L)GT(/A/S) in TM3, MPHNL(/V/F)F(/Y)LHS in TM6, and RS(/L/C/V)A(/S)IV(/T/I)P in TM9 (Figure 5). In conclusion, these results indicate that the NRAMP protein structure of tomato is highly conserved and that the rigorously conserved residues may play an important role in the transport of metal ions in NRAMP proteins.

### 2.5. Cis-Element and Promoter Analysis of the SlNRAMP Family Genes

**Figure 6:**
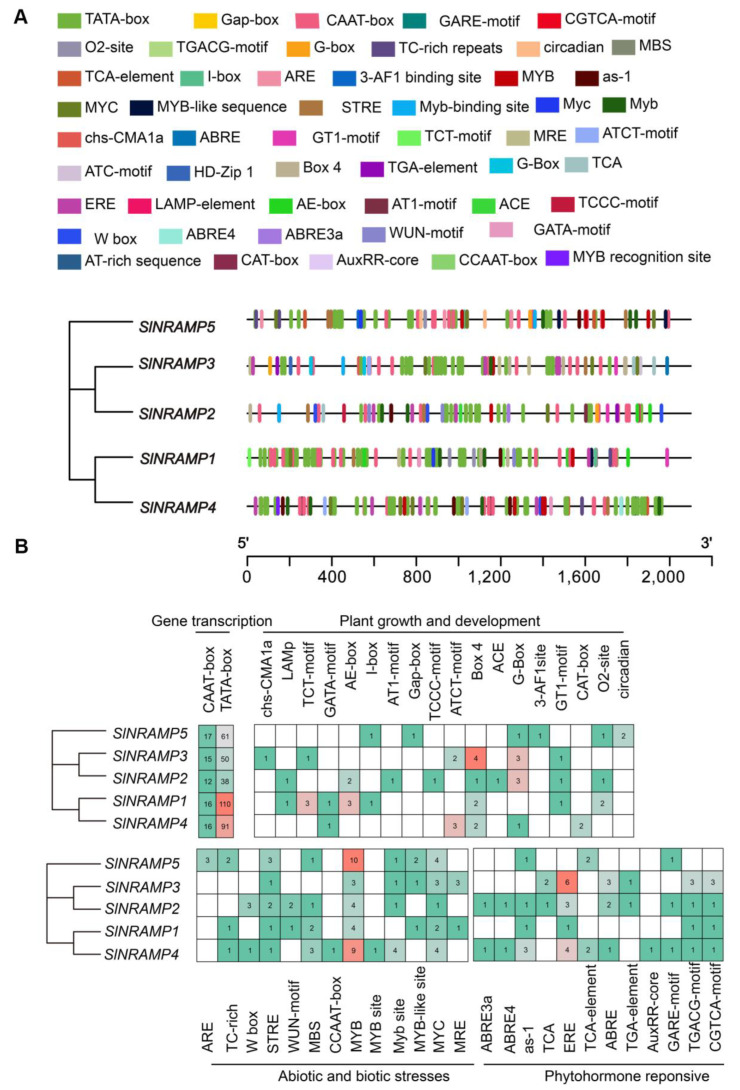
Cis-element number analysis of SlNRAMP family genes in tomato. The different intensity colors and numbers of the grid indicate the numbers of different promoter elements in the SlNRAMP family genes (**A**); the different-colored histogram represents the sum of the cis-acting elements (**B**).

To gain a deeper understanding of the possible regulatory mechanisms of tomato NRAMP genes, we identified 26 cis-acting elements within the 2 kb promoter region sequence upstream of the start codon of the SlNRAMP family genes. These elements were primarily related to gene transcription, plant growth and development, abiotic and biotic stress, and phytohormone response elements (Figure 6 and Table S6).

The promoter regions of all genes contained core elements, such as the CAAT-box and TATA-box. The number of CAAT-boxes ranged from 12 to 17, while the number of TATA-boxes (ATTAAT) ranged from 38 to 110, and the number of Box-4 elements (TAC-GTG) ranged from 1 to 12 (Figure 6 and Table S6). Among the cis-acting regulatory elements in the plant growth and development group, about 85% were involved in light response, mainly including motifs, such as ATCT-motif, AE-box, Box-4, and G-box. Other interesting motifs were also observed, such as circadian control and tissue-specific motifs, such as CAT-box (GCCACT) for meristem and O2-site (GT(/A)TGAT(/C)GTGA(/G)) for zein metabolism regulation (Figure 6 and Table S6).

In the abiotic and biotic stress category, different elements associated with stress responses, such as wounding, oxidation, heat, defense, and drought, were observed. Among them, most elements corresponded to two general transcription factor binding site class motifs: 43 MYB-binding elements ((C(/T)AACC(/A/T)A(/G))) and 14 MYC-binding elements (C(T)AT(/A/C)TTG(/A)). In addition, other stress-specific cis-elements were identified (Figure 6 and Table S6). Two of them were responsive to wounding and pathogens, including the W-box and WUN-motif, and the TC-rich repeat, STRE, was related to high temperature, drought (such as MBS), and anaerobic conditions (such as the ARE-motif) (Figure 6 and Table S6).

The phytohormone response elements group showed that a relatively high quantity of hormone response elements was linked to abscisic acid, ethylene, and methyl-jasmonate (MeJA) responsiveness, comprising 18, 22, and 25% of the total, respectively (Figure 6B). Specifically, ABRE3a, ABRE4, and ABRE elements were identified as being involved in abscisic acid responsiveness, while the TGACG- and CGTCA-motifs were associated with MeJA responsiveness (Figure 6 and Table S6). Additionally, ERE was involved in the ethylene response. The TCA element ((T(/C/A)ATCTTT(/C)T(/A)T)) was involved in salicylic acid responsiveness, while the as-1 element played a role in both salicylic acid and oxidative stress responsiveness (Figure 6 and Table S6). Moreover, the GARE-motif was linked to gibberellin responsiveness, while both the TGA element and the AuxRR core were involved in auxin responsiveness (Figure 6 and Table S6).

### 2.6. Expression Profiles of the SlNRAMP Family Genes in Response to Nutrient Deficiency in Tomato

The tissue-specific expression profiles of SlNRAMPs were investigated in various tomato tissues at ripe stages using qRT-PCR. Transcripts of all SlNRMAP genes, except for SlNRMAP1, were detected in specific tissues with distinct and partially overlapping expression patterns (Figure S2). *SlNRMAP2* showed lower expression levels compared to *SlNRAMP3*, *SlNRAMP*4, and *SlNRAMP*5, with relatively higher expression in flowers. *SlNRAMP3* and *SlNRMAP4* exhibited higher expression in leaves and flowers but lower expression in roots, pulp, and seeds. In contrast, *SlNRMAP5* showed relatively higher expression levels in roots and could be detected in all tissues.

**Figure 7:**
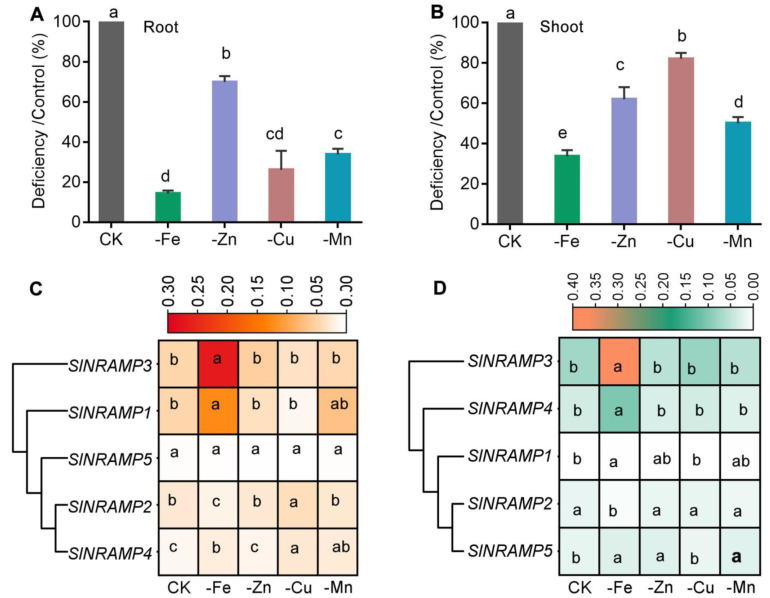
Expression levels of SlNRAMP family genes in tomato were subjected to Fe, Cu, Mn, and Zn deficiency. The 14-day-old seedlings were subjected to four nutrient deficiencies (-Fe, -Cu, -Zn, and -Mn) for 7 days, and then the shoots and roots elements concentration, as well as SlNRMAP gene family expression levels, were determined. The accumulation (%) of these elements in roots (**A**) and shoots (**B**). Roots (**C**) and leaves (**D**) were separately harvested for quantitative real-time polymerase chain reaction (qRT-PCR) analysis. The gene expression level was normalized against the reference gene *SlActin* using 2^(−∆Ct)^ values. Data (means ± SE, n = 5) sharing the same letter(s) above the error bars are not significantly different at the 0.05 level based on Duncan‘s test. Because the value of the standard error is a bit large for some gene expression levels, there is no significantly difference between them by statistical analysis, so the cells with different color were having the same letter.

To further explore the potential functions of tomato NRAMP genes in maintaining nutrient balance, SlNRAMPs were examined for change in expression in response to four nutrient deficiencies at the seedling stage. As shown in Figure 7A,B and Figure S3, deficiency of the essential nutrient elements, including Fe, Cu, Mn, and Zn, inhibited the growth and led to the decrement of the concentrations of these elements in shoots. Under normal conditions, the SlNRAMPs were expressed in both roots and leaves, with *SlNRAMP1* having the highest expression level in roots and *SlNRAMP3* having the highest expression level in leaves. Interestingly, the gene expression of *SlNRAMP3* and *SlNRAMP4* in roots and leaves, as well as the gene expression of *SlNRAMP5* in leaves and *SlNRAMP1* in roots, was strongly induced and upregulated by Fe deficiency. However, the gene expression of *SlNRAMP2* in the roots and leaves was strongly suppressed by Fe deficiency compared to the control (Figure 7C,D and Figure S4, Table S7). In the case of the Zn deficiency treatment, there was a significant enhancement in the gene expression of *SlNRAMP5* in the leaves compared to the control. However, no significant changes were observed in the gene expression of other family members. Cu deficiency led to a significant increase in the expression of *SlNRAMP4* genes in roots, while the expression levels of the other family members remained significantly unaffected. Last, under Mn deficiency treatment, there was a significant increase in the gene expression level of *SlNRAMP4* in the roots and a significant decrease in *SlNRAMP2* in leaves compared to the control. However, the expression levels of the other family members remained significantly unchanged.

### 2.7. Expression Analysis of the SlNRAMP Family Genes in Response to AMF Colonization under Cd Stress

**Figure 8:**
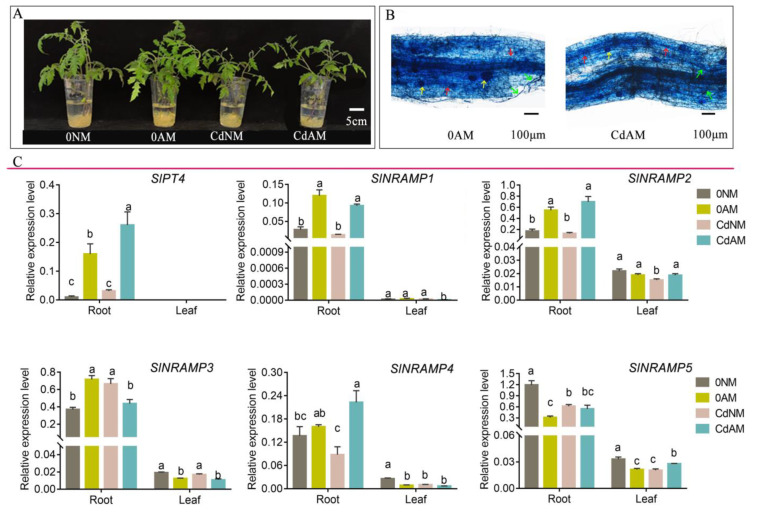
Expression levels of the SlNRAMP family genes in response to Cd stress during AMF symbiosis. The 14-day-old seedlings were inoculated with or without AMF under Cd stress for 35 days, and then the shoots and roots Cd/P concentration, as well as SlNRMAP gene family expression levels, were determined. Photograph of the treatments (**A**). Visualization of mycorrhizal fungal structures in mycorrhizal roots grown with or without Cd stress (**B**). Red arrows indicate arbuscules, yellow arrows indicate vesicles, and green arrows indicate hyphae. Roots and leaves were harvested separately for qRT-PCR analysis (**C**). The gene expression level was normalized against the reference gene *SlActin* using 2^(−∆Ct)^ values. Data (means ± SE, n = 5) sharing the same letter(s) above the error bars are not significantly different at the 0.05 level, based on Duncan’s test.

To further investigate the response of tomato SlNRAMPs to Cd stress and AMF, we conducted qRT-PCR analysis to examine the expression patterns of SlNRAMPs under Cd stress, with or without AMF. As illustrated in Figure 8, Arbuscular mycorrhizal fungi structures were both observed in AMF-inoculated tomato roots with or without Cd addition, including the hyphae, arbuscules, and vesicles (Figure 8A,B and Figure S5). Furthermore, the *SlPT4* gene, regarded as a marker gene to indicate the extent of AMF infection, was strongly induced by AMF and expressed exclusively in the roots, regardless of the presence or absence of Cd (Figure 8C). This suggests that tomato plants can establish a more beneficial symbiotic relationship with AMF under Cd stress.

Under normal growth conditions, expression of the *SlNRAMP1*, *SlNRAMP2,* and *SlNRAMP3* genes in the roots was strongly induced and upregulated by AMF, while the expression of *SlNRAMP5* in the roots and leaves, as well as *SlNRAMP3* and *SlNRAMP4* in the leaves, was strongly inhibited by AMF (Figure 8C). Interestingly, compared to Cd NM treatments, *SlNRAMP3* in the roots and leaves were strongly downregulated by AMF under Cd AM treatment; however, *SlNRAMP1*, *SlNRAMP2,* and *SlNRAMP4* in the roots were strongly activated in the roots colonized by AMF (Figure 8C).

### 2.8. P and Cd Concentration in Tomato Root and Shoot Altered by AMF Colonization under Cd Stress

Compared to 0 NM treatment, Arbuscular mycorrhizal fungi inoculation significantly promoted P concentration in tomato roots and shoots by 60% and 50% under 0 AM treatment, respectively. Under Cd stress, the P concentration of shoots and roots increased by approximately 25% and 29% when compared to those of nonmycorrhizal plants (Table 2), respectively, suggesting that AMF colonization and Cd toxicity affect the P homeostasis. Furthermore, Arbuscular mycorrhizal fungi inoculation significantly reduced Cd concentrations in tomato roots and shoots by 40% and 38% under Cd stress.

## 3. Discussion

The genome-wide identification of NRAMP proteins was conducted in different plant species; however, the NRAMP protein family in tomato has yet to be fully explored. Using bioinformatics analysis, only five NRAMP protein genes in tomato were noted in this study, which was similar to watermelon (*Citrullus lanatus*) (4) [47] and potato (5) [10] but generally lower than for many plant species, including peanut (*Arachis hypogaea* L.) (15) [9], thale cress (6) [14], black cottonwood (11) [11], rice (7) [4], common bean (*Phaseolus vulgaris* L.) (7) [48], and tea (*Camellia sinensis*) (11) [49].

Phylogenetic analysis of the NRAMP genes revealed that these genes could be classified into two subfamilies: subfamily I and subfamily II. This classification aligned with the classifications found in previous studies [8,48,50]. The basal cluster of NRAMP genes consisted of genes from potato and tomato, indicating that a single ancestral NRAMP gene underwent independent duplications in solanaceous lineages after the Solanaceae family split. Subfamily I is composed of the two SlNRAMP proteins (SlNRAMP2/3), while subfamily II includes two other SlNRAMP members (SlNRAMP1/4). These subfamilies differ in various characteristics, such as the PI, composition of conserved motifs, subcellular localization of the SlNRAMP proteins, and exon/intron structure. However, within each subfamily, these traits are highly conserved [4,14]. In subfamily I, SlNRAMP proteins are acidic, with a PI ranging from 5.23 to 5.61, and share nine conserved motifs (motifs 1 to 9). In contrast, subfamily II members are basic proteins, with a PI ranging from 7.15 to 8.85, and share ten motifs (Table 1 and Table S2, Figure 2A). The exon/intron structure differs between the two subfamilies, with subfamily I genes having 4 exons and subfamily II genes having 13 exons (except for SlNRAMP5, which has 7 exons (Figure 2C). The subcellular localization of the SlNRAMP proteins also varies between the subfamilies [4,8,20,51]. Subfamily I proteins are predicted to be localized in the vacuole membrane, while subfamily II proteins are localized in the plasma membrane except for SlNRAMP4, which is localized to both the vacuole membrane and the plasma membrane (Table 1). Similar localization patterns have been observed in other plants; for example, subfamily I members from rice (OsNRAMP2) [16], thale cress (AtNRAMP3/4) [6,14], and soybean (GmNRAMP1a/GmNRAMP2a/GmNRAMP2b/GmNRAMP3a) [42] are localized in the tonoplast, while some subfamily II members (OsNRAMP3/4/5, AtNRAMP1, and GmNRAMP5a/GmNRAMP7) are localized in the plasma membrane [8,13,52]. The classification of NRAMP genes into subfamilies suggests close genetic conservation among NRAMPs, with each subfamily containing members of different species.

The conserved motifs of NRAMP genes within the same group imply their functional similarity and evolutionary relationship. In this study, SlNRAMP proteins were found to contain 9–12 transmembrane regions (Table 1, Figure S1), similar to rice [4], potato [10], and thale cress (10–12 transmembrane domains) [13,53,54]. However, SlNRAMP consisted of 530–1316 amino acid residues, which was much higher than rice [4], potato [10], and thale cress (about 500 amino acid residues) [55]. This discrepancy may be due to a broken NRAMP domain in tomato or species variation. Moreover, the consensus residues between transmembrane domains 8 and 9 have been reported in several NRAMP proteins, such as AtNRAMPs in thale cress [6], PvNRAMPs in common bean [48], and StNRAMPs in potato [10]. These consensus residues play a crucial role in protein function. In tomato, SlNRAMP proteins also carried similar consensus residues, except for SlNRAMP5, which contained other residues (Figure 5).

The presence of conserved motifs and the distribution of motifs within different groups of NRAMP genes provide insights into their evolutionary relationships and potential functional roles. Based on conserved motif and gene structure (exon/intron) analyses, most SlNRAMP proteins contained nine motifs that were distributed similarly among genes on the same branch of the evolutionary tree in tomato (Figure 2, Table S2). For example, SlNRAMP1 and SlNRAMP4 shared the same motif compositions, SlNRAMP2 and SlNRAMP3 shared nine same motifs, and SlNRAMP5 had the lowest number of motifs, suggesting that different motif distributions in different groups may contribute to functional diversity during the evolutionary process. Similar observations were also found in rice [4], tea [49], cucumber [47], and peanut [9].

The expansion and evolution of gene families rely on various duplication events, such as whole genome, segmental, tandem, and gene duplication events [56]. These duplication events not only lead to the emergence of new functions but also result in functional redundancy [57]. Previous studies have identified different types of duplication events in several species, such as cacao [58], peanut [9], rice [4], and soybean [59]. However, no tandem or segmental duplications were found in tomato in this study, except for genomic collinearity events (Figure 3A, Table S3). Similar results have been reported in the NRAMP gene families in duckmeat [60], which may be attributed to the number of SlNRAMP genes and the level of conservation. To explore the evolutionary relationship of NRAMP genes, we conducted genomic collinearity analysis of five species and identified numerous colinear gene pairs (Figure 3B, Table S4). The presence of continuous collinear gene pairs in these five species suggested that the NRAMP genes were highly conserved. All SlNRAMP proteins were successfully modeled using the 3D model template EcoDMT, belong to the SLC11 (NRAMP) family, transporting divalent transition-metal ions such as Fe^2+^, Mn^2+^, and Cd^2+^ across cellular membranes [61,62,63]. Previous studies have shown that AtNRAMP3 shares several highly conserved amino acid residues with EcoDMT, affecting the transport of Cd, Fe, and Mn through site-directed mutagenesis and metal toxicity growth assays in yeast [54]. Similarly, the SlNRAMP gene family exhibited high conservation when compared to EcoDMT. The sequence GQSSTITGTYAGQY(/F)V(/I)MQ(/E)GFL(/I), located in TM8, TM9 [64], and the region between them, is highly conserved in SlNRAMP1–4, and EcoDMT. However, some variations were also noted in the SlNRAMP5 protein sequence. Similar findings have been reported for AhNRAMP1.2, CsNRAMP3, and CsNRAMP8 of the other gene families in peanut [9] and tea [49]. Additionally, several highly conserved amino acid residues are identified in different TMDs with sequence identities, including DPGN(K) in TM1, DI(/L)Q(/P/T/A)E(/Q/M)VI(/L)GT(/A/S) in TM3, MPHNL(/V/F)F(/Y)LHS in TM6, and RS(/L/C/V)A(/S)IV(/T/I)P in TM9 (Figure 4 and Figure 5). The structural similarities between SlNRAMPs and EcoDMT suggest that they may have similar physiological functions.

Gene family members exhibit various expression patterns in response to changes in the external environment. Analyzing cis-acting elements in gene promoter regions is crucial for understanding the regulation of gene expression. In the present study, we identified 26 types of cis-acting elements and categorized them into four groups (Figure 6 and Table S6). Within the plant growth and development group, 85% of the elements were involved in light response, including the ATCT-motif, AE-box, Box-4, and G-box. Other motifs, such as CAT-box (GCCACT), were associated with regulating meristem and O_2_-site zein metabolism. Moreover, the abiotic and biotic stress group was the most abundant, consisting of 13 types. The MYB family and the MYC family are relatively the most numerous in this group, with MYB being the second-largest transcription factor family in eukaryotes and being involved in various biological processes [65,66]. Studies have demonstrated that overexpressing *MYB12* and *MYB75* in transgenic plants greatly enhances the production of flavonoids, which possess strong antioxidant properties. This enhanced flavonoid accumulation improves the plants’ resilience to abiotic stresses like drought and oxidative stress. Furthermore, all family members also possess abundant MYC elements. Moreover, the MYC family belongs to the bHLH transcription factor superfamily and plays a significant role in plant growth and development, particularly in enhancing stress resistance [67]. Plant hormones play crucial roles in regulating NRAMP gene expression. For instance, the expression of rice *OsNRAMP1* is inhibited by abscisic acid and MeJA, while the expression levels of *OsNRAMP2/3* can be activated and upregulated [68]. In tomato, the promoter regions of SlNRAMP gene family members contain ethylene, salicylic acid, and MeJA regulatory elements, specifically as-1, ERE, TGACG-motif, and CGTCA-motif, suggesting that the regulation of the tomato SlNRAMP family genes are intricately controlled by a complex regulatory network.

NRAMP genes play multiple roles in transporting various metal ions in plants, especially Fe [14,69]. It was concluded that Fe deficiency significantly induced the expression of *AtNRAMP1*, *AtNRAMP6*, *AtNRAMP3,* and *AtNRAMP4* in thale cress [6,13,14], *OsNRAMP5* in rice [52], and *SlNRAMP1*, *SlNRAMP3* in tomato [46] (Figure 7 and Table S7). The present study concurred with a previous study that the depletion of Fe significantly increased the gene expression of *SlNRAMP1*, *SlNRAMP3,* and *SlNRAMP4* in tomato roots and leaves. Interestingly, *SlNRAMP2* may have a different function in Fe homeostasis since the expression of this gene in both the roots and leaves was significantly reduced under Fe deficiency. A similar result was reported, whereby *AtNRAMP2* gene expression in thale cress was downregulated under Fe-deficient conditions [13].

NRAMP genes are also known to transport a range of divalent transition metals, including Mn^2+^, Cu^2+^, and Zn^2+^ [70,71]. However, only *SlNRAMP5* in leaves showed significant upregulation in response to Zn deficiency, while the gene expression of other family members remained unchanged (Figure 7), as has been noted in *StNRAMP3* in potato [10]. Under Cu deficiency, both *SlNRAMP2* and *SlNRAMP4* were highly upregulated in roots, while *SlNRAMP4* in the roots and *SlNRAMP5* were both significantly enhanced by Mn deficiency. A previous study found that *AtNRAMP1* is an Mn transporter with a high affinity for Mn under low Mn conditions [61,62]. *SlNRAMP4* may have functions similar to *AtNRAMP1* due to its close evolutionary relationship.

Furthermore, NRAMP genes have been shown to affect the intracellular remobilization of Cd, leading to increased Cd tolerance in plants [9,51]. In this study, the expression of *SlNRAMP3* in roots was significantly increased under Cd stress, similar to the expression patterns of their orthologs from potato (*StNRAMP2*) [10,20], wheat (*Triticum polonicum* L.) (*TpNRAMP5*) [72], and rice (*OsNRAMP1*) [73]. It has been reported that *OsNRAMP1* and *OsNRAMP5* are involved in Cd uptake through roots, and knocking out both genes results in a significant decrease in Cd uptake compared to knocking out either of the genes alone [51,73]. This suggests that *SlNRAMP3*, *StNRAMP2,* and *OsNRAMP5* may have similar functions due to their location in the same subtribe and their involvement in Cd accumulation. However, the expression of *SlNRAMP5* in the leaves was significantly decreased under Cd exposure (Figure 8). Similar differential responses to Cd stress have been observed in other plants. For example, in rice, the expression of *OsNRAMP1* in roots and leaves was enhanced under Cd treatment, while the expression of *OsNRAMP5* in roots was inhibited, with little effect on the expression of *OsNRAMP2* [73]. These findings suggest that the SlNRAMP gene family has undergone functional differentiation during evolution and that different genes within the family exhibit different responses to different heavy metal stressors and in different tissues.

It has been well documented that mycorrhization in plant roots can restrict Cd invasion [30,70], as observed in rice [74], kenaf (*Hibiscus cannabinus* L.) [75], and poplar (*Populus yunnanensis*) [76]. However, the detailed related mechanism in tomato is not yet well understood. Our results indicate that AMF significantly reduced the uptake and accumulation of Cd in mycorrhizal tomato under Cd stress compared to uninoculated plants. Similar to the result reported by Wang et al. and Pan et al. [75,76], the Cd concentration was about 60% lower in the mycorrhizal-treated roots than in non-colonized roots under 50 mg/kg Cd treatment. Moreover, we found that AMF colonization and Cd toxicity affect the P homeostasis in tomato, as Cd stress inhibited the P uptake, while inoculation with AMF promoted the P uptake, which was consistent with previous research (Table 2). For instance, the AMF increased macro-elements (P in *Solanum nigrum* [77] and Ca, K, and Mg in maize (*Zea mays*) [78]) as well as trace elements (Zn and Cu in *alfalfa* [76]) under metal-stressed treatments. These results illustrate that there is a significant interaction between the co-existence of metals and nutrient uptake during AMF symbiosis. Thus, the mycorrhizal symbiosis between AMF and their host plants seems to form a protective strategy through the regulation of ion balance by AMF for heavy metal stress [29,30,76]. Furthermore, earlier studies found that AMF colonization in roots decreased *SlNRAMP1* and *SlNRAMP3* gene transcripts, specifically in plants grown in Breinigerberg soil rather than under Cd stress conditions [79]. However, in the current study, SlNRAMP3 was strongly inhibited by AMF in both Cd-treated roots and leaves, indicating that AMF restrained Cd-induced NRAMP expression in tomato. This may be due to the sequestration of Cd by fungal hyphae lowering Cd translocation into plants, hence decreasing the Cd-triggering effect on NRAMP expression [75,76]. Similarly, the inhibiting effect on gene expression by AM has been displayed in other Cd transporters in plants, such as *PsMTA* in peas [36] and *OsNRAMP5* and *OsHMA3* in rice [34]. These results suggested that the inhibiting effect of *SlNRMAP3* by AMF in tomato might be, at least partially, responsible for the reduced Cd accumulation in mycorrhizal plants under Cd stress. However, the relevant regulatory mechanisms still need to be further unveiled.

It is well established that AMF symbiosis improves the nutritional status of plants under different mineral deficiencies. Recently, it was found that Fe and Zn transporters (*HaIRT1*, *HaNRAMP1*, and *HaZIP1*) were concurrently involved with AMF-mediated alleviation of Fe deficiency in sunflower [80]. Similarly, upregulation of *MtZIP6* [81] and *MtZIP14* [82] induced by AMF inoculation led to increasing Zn uptake for Zn balance in *alfalfa* (*Medicago sativa* L.) at low Zn condition. In this study, the expression of *SlNRAMP1*, *SlNRAMP2,* and *SlNRAMP3 were not only* regulated by AMF inoculation but also responded positively to Cu, Zn, and Mn deficiency. Therefore, AM symbiosis might contribute to these nutrients’ uptake by regulating the NRAMP genes. These findings herein provide valuable information for further studies on the physiological roles of SlNRAMPs in the uptake of Cd and mineral nutrients during AM symbiosis.

## 4. Materials and Methods

### 4.1. Plant Materials and Growth Conditions

Tomato (*Solanum lycopersicum* L. cv hezuo903) was purchased from seed stores. After being surface sterilized, the seeds were germinated for one week under the following conditions: 16 h light at 28 °C and 8 h dark at 18 °C; the relative humidity was controlled at 60% to 70%. The seedlings were then transplanted to quartz sand for one week with half Hoagland solution; the culture conditions and nutrient solutions were followed as described previously by Liu et al. (2020) [26,83]. The 14-day-old seedlings were transplanted and grown in a greenhouse for four months before separately harvesting roots, leaves, flowers, pulp, and seed for further RNA extraction and qRT-PCR analysis.

In order to explore the potential reactions of *SlNRAMPs* to nutrient deficiency, the plants were subsequently transferred to a pot culture to continue growing under the specified treatments. The 14-day-old seedlings were subjected to four nutrient deficiencies (-Fe, -Cu, -Zn, and -Mn) for 7 days. Each treatment had five biological replicates. During the growing period, nutrient solutions were renewed once every two days. Both young leaves and roots were separately sampled for further RNA extraction and qRT-PCR analysis.

The 14-day seedlings were divided into four groups: mycorrhizal plants (AM) were inoculated with approximately 200 spores of the AMF (*Rhizophagus irregularis*) [26], which served as an AM group. As an NM group, nonmycorrhizal plants (NM) were obtained by inoculating with autoclaved inoculum. Both the NM and AM groups were then grown in sterilized sand containing a nutrient solution with either 0 or 100 µM Cd. The composition of the nutrient solution was similar to the one mentioned earlier, except for the addition of 25 μM Pi to ensure mycorrhizal colonization. This resulted in four treatment groups: 0 NM, 0 AM, Cd NM, and CdAM, each with five replicates. Throughout the growth period, the nutrient solutions were refreshed weekly. After a treatment period of 35 days, both young leaves and roots were separately sampled for further RNA extraction and qRT-PCR analysis.

### 4.2. Identification and Sequence Analysis of the NRAMP Genes Family in Tomato

To identify tomato SlNRAMP genes, the protein sequences of *Arabidopsis thaliana* (6 genes) and rice (7 genes) were obtained from phytozome1 and used as queries for TBLASTP against tomato genome proteins (ITAG release 4.0 https://solgenomics.net/tools/blast/ (accessed on 22 March 2023)). In addition, NRAMP family proteins were further identified using the Hidden Markov Model (HMM) profile of the NRAMP domain (PF01566) with the HMM search tool (https://www.ebi.ac.uk/Tools/hmmer/search/hmmscan, accessed on 22 March 2023). Then, putative NRAMP proteins were filtered by checking the presence of a conserved NRAMP domain (IPR001046) using the Pfam tool (https://www.ebi.ac.uk/interpro/search/sequence/, accessed on 22 March 2023). Transmembrane helices in proteins were predicted using the TMHMM Server v. 2.0 (http://www.cbs.dtu.dk/services/TMHMM/, accessed on 22 March 2023).

### 4.3. Physicochemical Properties and Structure Characteristics of SlNRAMP Proteins

The molecular weight (MW) and PI (isoelectric points) were determined using the ProtParam tool (http://web.expasy.org/protparam, accessed on 22 March 2023). The prediction of subcellular localization for NRAMPs was performed using ProtComp version 9.0 online tool (http://linux1.softberry.com/berry.phtml?group=programs&subgroup=proloc&topic=protcomppl, accessed on 22 March 2023).

Gene structures were generated by downloading CDSs and genomic sequences of tomato NRAMP genes on the Gene Structure Display Server 2.0 online tool (http://gsds.cbi.pku.edu.cn/index.php, accessed on 22 March 2023). Finally, the conserved motif, transmembrane structure, and conserved domain of the tomato NRAMP gene family were visualized using TBtools [84]. All SlNRAMP protein sequences in tomato were utilized as queries against the SWISS-MODEL template library SMTL to identify suitable templates for building their 3D structure. The templates were selected based on their global model quality estimate (GMQE) [85] and QMEANDisCo global score [86] (Table S5).

### 4.4. Chromosomal Locations and Duplication of NRAMP Genes

The chromosomal locations and gene collinearity of NRAMP genes were analyzed and visualized via the One-Step MCScanX, Dual Synteny plot, and Dual Synteny plot for MCscanx of TBtools software. The collinearity relationship of SlNRAMP genes between the tomato genome and the genomes of other plants (thale cress, potato, rice, alfalfa, and soybean) was analyzed via the One-Step MCScanX integrated into TBtools software v. 1. 108 [86]. Gene collinearity was analyzed using the One-Step MCScanX in TBtools software v. 1. 108 [87].

### 4.5. Cis-Acting Regulatory Elements and MicroRNA Target Sites of NRAMP Genes

The PlantCARE online tool (http://bioinformatics.psb.ugent.be/webtools/plantcare/html/ (accessed on 22 March 2023)) was used to analyze the ATG 2 kb upstream sequence of the promoter sequence from the tomato NRAMP family gene.

### 4.6. Phylogenetic Analysis

The NRAMP protein sequences of tomato, thale cress, rice, soybean, alfalfa, and potato were aligned by ClustalW in MEGA 6.0 (version 10.2.6). The aligned files were used to construct a phylogenetic tree using the maximum likelihood method based on the Poisson model, with 1000 bootstrap replicates. The phylogenetic tree was displayed and modified using ITol (https://itol.embl.de/tree/1121799238308031679449237, accessed on 22 March 2023).

### 4.7. cDNA Preparation and qRT-PCR Analysis

To investigate the expression levels of SlNRAMP genes in tomato across different tissues and in response to various treatments, we performed cDNA synthesis using approximately 2 μg of DNase-treated total RNA with a reverse transcription kit. The resulting cDNAs were then utilized in subsequent qRT-PCR reactions. These reactions were carried out using the SYBR premix ExTaq kit on an Applied Biosystems Plus real-time PCR system. Each reaction consisted of 10 μL of SYBR Green premix, 0.2 μM of gene-specific primers, and 1 μL of the cDNA template at a final volume of 20 μL. The PCR protocol involved an initial incubation at 95 °C for 15 s, followed by 40 cycles of 95 °C for 5 s and 68 °C for 30 s. Additionally, a final cycle for dissociation curves was conducted to validate the specificity of the amplification. To normalize the transcription levels of each target gene, we used the constitutive *SlActin* gene of tomato as reference genes. Both the constitutive *SlActin* gene and *SlEF1α* (elongation factor 1α) of tomato were used as reference genes. Relative gene expression was calculated by the formula 2^(−∆Ct)^ (Target gene relative expression levels = (Power (2, − (Ct_target gene_ − Ct_internal reference gene_ Mean)), Ct: computerized tomography). Different target gene expressions were normalized against the same internal reference gene. The primer sequence for qRT-PCR in this study can be found in Table S8.

### 4.8. Visualization of Mycorrhizal Fungal Structures

To visualize the fungus, the plant roots were washed with deionized water and separated randomly into segments (1 to 2 cm). They were then treated with 10% KOH for 1 h at 90 °C, washed with water and acidified with 5% (*v*/*v*) HCl solution for 8 min at 25 °C, and stained with 0.2% (*w*/*v*) trypan blue solution at 90 °C for 1 h [83]. Finally, the root segments were observed with a light microscope (OLYMPUS DP74, Tokyo, Japan).

### 4.9. Plant Elements Concentration

The plants were harvested, and the shoots and roots were separated, dried at 70 °C to a constant weight, ground into a powder, and stored at room temperature before analysis. The sample was subsequently digested in HNO_3_ at 160 °C for 12 h in a microwave oven (MARS-240, USA CEM, Matthews, NC, USA) [55]. The concentration of Cd, Fe, Cu, Zn, and Mn was measured using inductively coupled plasma mass spectrometry (ICP-MS; 7700, Agilent, Palo Alto, CA, USA). The total P concentration was measured by the molybdate blue method and was performed as described previously by Chen, Hu, Sun, and Xu [44]. Deficiency/Control (%) was calculated by the following formula:

Deficiency/Control (%) in roots =RC1_X_/RC2_x_

Deficiency/Control (%) in shoots =SC1_X_/SC2_x_

R: roots; S: shoots; C1: element (Fe, Zn, Cu, and Mn, respectively) concentration in roots or shoots under different deficiency; C2: the average concentration of the same element in roots or shoots under control; X: Fe, Zn, Cu, and Mn, respectively.

### 4.10. Statistical Analysis

The experimental data were calculated using GraphPad Software 7.0 (shown as the mean ± standard error), followed by statistical analysis using SPSS 20.0. The means were subjected to a test of statistical significance using Duncan’s test (*p* < 0.05).

## 5. Conclusions

By conducting genome-wide searches of available databases, we identified five NRAMP genes in tomato. Further characterization of these genes in phylogenetic evolution and transcriptional regulation profiles revealed the evolutionary conservation and functional divergence of the tomato NRAMP family in response to different nutrient deficiencies and Cd stress, with or without AMF. The expression of NRAMP genes was differently influenced by nutrient deficiencies, Cd stress, and AMF. Specifically, under Cd stress, the expression of *SlNRAMP3* significantly increased in the roots, while *SlNRAMP5* expression was inhibited. Interestingly, the presence of AMF resulted in opposite effects compared to non-inoculated plants under Cd stress. Furthermore, AMF significantly inhibits the uptake and accumulation of Cd in mycorrhizal tomato under Cd stress. Hence, these findings provide valuable genetic information for improving tomato resilience to nutrient deficiency and heavy metal stress by developing AM symbiosis.

## Figures and Tables

**Table 1:** Molecular characterization of SlNRAMP family genes and the corresponding proteins in tomato.

Gene Name	SlNRAMP1	SlNRAMP2	SlNRAMP3	SlNRAMP4	SlNRAMP5
Gene ID	Solyc11g018530.2.1	Solyc04g078250.3.1	Solyc02g092800.3.1	Solyc03g116900.3.1	Solyc09g007870.3.1
Genomic location	8,645,242–8,648,856	63,109,871–63,113,592	54,402,272–54,405,963	67,650,010–67,659,122	1,399,607–1,407,814
Gene length (bp)	1853	2128	2210	1984	4841
CDS (bp)	1593	1560	1347	1602	3951
AA	530	519	509	522	1316
MW (kDa)	57.78	57.15	56.15	56.71	142.67
PI	7.15	5.23	5.61	8.85	6.03
Instability	31.99	48.13	29.73	35.21	40.57
Aliphatic index	124.55	111.43	119.35	121.26	88.76
Gravy	0.6	0.357	0.581	0.509	−0.013
TN	12	9	11	12	11
SL	PM	V	V	V, PM	PM

TN: transmembrane regions; SL: subcellular localization; PM: plasma membrane; V: vacuole membrane; PI: isoelectric points.

**Table 2:** The Cd and P concentrations of tomato roots and shoots in the presence or absence of AM fungal colonization under Cd stress.

Treatments	Cd Concentration (mg/g)		P Concentration (mg/g)	
	Root	Shoot	Root	Shoot
0 NM	0.004 ± 0 c	0.001 ± 0 c	1.446 ± 0.068 bc	0.755 ± 0.006 b
0 AM	0.003 ± 0.001 c	0.001 ± 0 c	2.288 ± 0.074 a	1.129 ± 0.075 a
100 NM	3.473 ± 0.530 a	0.118 ± 0.013 a	1.199 ± 0.046 c	0.782 ± 0.015 b
100 AM	2.073 ± 0.236 b	0.073 ± 0.007 b	1.544 ± 0.121 b	0.977 ± 0.061 a

The 14-day-old seedlings were inoculated with or without AMF under Cd stress for 35 days, and then the shoots and roots Cd/P concentrations were determined. Data (means ± SE, n = 3). Different letters indicate significant differences (*p* < 0.05, Duncan’s test).

## Data Availability

The data presented in this study are available on request from the corresponding author upon reasonable request.

## Supplementary Materials

The supporting information can be downloaded at: https://www.mdpi.com/article/10.3390/ijms25158269/s1.

## Abbreviations

AMF	Arbuscular mycorrhizal fungi
Cu	Copper
Cd	Cadmium
CDSs	Coding sequences
GMQE	Global model quality estimate
Fe	Iron
Mn	Manganese
MeJA	Methyl-jasmonate
NRAMP	Natural resistance-associated macrophage protein
PI	Isoelectric points
qRT-PCR	Quantitative real-time polymerase chain reaction
Zn	Zinc

## References

[1] Bozzi A.T., Gaudet R. (2021). Molecular Mechanism of Nramp-Family Transition Metal Transport. J. Mol. Biol..

[2] Courville P., Chaloupka R., Cellier M.F.M. (2006). Recent Progress in Structure-Function Analyses of Nramp Proton-Dependent Metal-Ion Transporters. Biochem. Cell Biol..

[3] Nevo Y., Nelson N. (2006). The NRAMP Family of Metal-Ion Transporters. Biochim. Biophys. Acta.

[4] Mani A., Sankaranarayanan K. (2018). In Silico Analysis of Natural Resistance-Associated Macrophage Protein (NRAMP) Family of Transporters in Rice. Protein J..

[5] Chen Y., Li G., Yang J., Zhao X., Sun Z., Hou H. (2021). Role of Nramp Transporter Genes of Spirodela Polyrhiza in Cadmium Accumulation. Ecotoxicol. Environ. Saf..

[6] Lanquar V., Ramos M.S., Lelièvre F., Barbier-Brygoo H., Krieger-Liszkay A., Krämer U., Thomine S. (2010). Export of Vacuolar Manganese by AtNRAMP3 and AtNRAMP4 Is Required for Optimal Photosynthesis and Growth under Manganese Deficiency. Plant Physiol..

[7] Kumar A., Singh G., Kumar R., Prasad B. (2022). Genome Wide Analysis and Identification of NRAMP Gene Family in Wheat (*Triticum aestivum* L.). Pharma Innov. J..

[8] Qin L., Han P., Chen L., Walk T.C., Li Y., Hu X., Xie L., Liao H., Liao X. (2017). Genome-Wide Identification and Expression Analysis of *NRAMP* Family Genes in Soybean (*Glycine Max* L.). Front. Plant Sci..

[9] Tan Z., Li J., Guan J., Wang C., Zhang Z., Shi G. (2023). Genome-Wide Identification and Expression Analysis Reveals Roles of the NRAMP Gene Family in Iron/Cadmium Interactions in Peanut. Int. J. Mol. Sci..

[10] Tian W., He G., Qin L., Li D., Meng L., Huang Y., He T. (2021). Genome-Wide Analysis of the NRAMP Gene Family in Potato (*Solanum tuberosum*): Identification, Expression Analysis and Response to Five Heavy Metals Stress. Ecotoxicol. Environ. Saf..

[11] Ma X., Yang H., Bu Y., Zhang Y., Sun N., Wu X., Jing Y. (2023). Genome-Wide Identification of the NRAMP Gene Family in Populus Trichocarpa and Their Function as Heavy Metal Transporters. Ecotoxicol. Environ. Saf..

[12] Zhang J., Zhang M., Song H., Zhao J., Shabala S., Tian S., Yang X. (2020). A Novel Plasma Membrane-Based NRAMP Transporter Contributes to Cd and Zn Hyperaccumulation in Sedum Alfredii Hance. Environ. Exp. Bot..

[13] Curie C., Alonso J.M., Le Jean M., Ecker J.R., Briat J.F. (2000). Involvement of NRAMP1 from *Arabidopsis thaliana* in Iron Transport. Biochem. J..

[14] Lanquar V., Lelièvre F., Bolte S., Hamès C., Alcon C., Neumann D., Vansuyt G., Curie C., Schröder A., Krämer U. (2005). Mobilization of Vacuolar Iron by AtNRAMP3 and AtNRAMP4 Is Essential for Seed Germination on Low Iron. EMBO J..

[15] Li J., Wang Y., Zheng L., Li Y., Zhou X., Li J., Gu D., Xu E., Lu Y., Chen X. (2019). The Intracellular Transporter AtNRAMP6 Is Involved in Fe Homeostasis in Arabidopsis. Front. Plant Sci..

[16] Chang J.-D., Xie Y., Zhang H., Zhang S., Zhao F.-J. (2022). The Vacuolar Transporter OsNRAMP2 Mediates Fe Remobilization during Germination and Affects Cd Distribution to Rice Grain. Plant Soil.

[17] Yang M., Zhang W., Dong H., Zhang Y., Lv K., Wang D., Lian X. (2013). OsNRAMP3 Is a Vascular Bundles-Specific Manganese Transporter That Is Responsible for Manganese Distribution in Rice. PLoS ONE.

[18] Ishimaru Y., Takahashi R., Bashir K., Shimo H., Senoura T., Sugimoto K., Ono K., Yano M., Ishikawa S., Arao T. (2012). Characterizing the Role of Rice NRAMP5 in Manganese, Iron and Cadmium Transport. Sci. Rep..

[19] Sasaki A., Yamaji N., Yokosho K., Ma J.F. (2012). Nramp5 Is a Major Transporter Responsible for Manganese and Cadmium Uptake in Rice. Plant Cell.

[20] Zhang Y., He T., Tian W., Xia Y., He Y., Su M., He G. (2023). The Expression of the StNRAMP2 Gene Determined the Accumulation of Cadmium in Different Tissues of Potato. Int. J. Mol. Sci..

[21] Guo J., Long L., Chen A., Dong X., Liu Z., Chen L., Wang J., Yuan L. (2022). Tonoplast-Localized Transporter ZmNRAMP2 Confers Root-to-Shoot Translocation of Manganese in Maize. Plant Physiol..

[22] Motaharpoor Z., Taheri H., Nadian H. (2019). Rhizophagus Irregularis Modulates Cadmium Uptake, Metal Transporter, and Chelator Gene Expression in Medicago Sativa. Mycorrhiza.

[23] Gutjahr C., Parniske M. (2013). Cell and Developmental Biology of Arbuscular Mycorrhiza Symbiosis. Annu. Rev. Cell Dev. Biol..

[24] Varga S., Soulsbury C.D. (2019). Arbuscular Mycorrhizal Fungi Change Host Plant DNA Methylation Systemically. Plant Biol..

[25] Marschner H., Dell B. (1994). Nutrient Uptake in Mycorrhizal Symbiosis. Plant Soil.

[26] Liu J., Chen J., Xie K., Tian Y., Yan A., Liu J., Huang Y., Wang S., Zhu Y., Chen A. (2020). A Mycorrhiza-Specific H^+^-ATPase Is Essential for Arbuscule Development and Symbiotic Phosphate and Nitrogen Uptake. Plant Cell Environ..

[27] Harrison M.J., Stacey G., Keen N.T. (1997). The Arbuscular Mycorrhizal Symbiosis. Plant-Microbe Interactions.

[28] Janeeshma E., Puthur J.T. (2020). Direct and Indirect Influence of Arbuscular Mycorrhizae on Enhancing Metal Tolerance of Plants. Arch. Microbiol..

[29] Riaz M., Kamran M., Fang Y., Wang Q., Cao H., Yang G., Deng L., Wang Y., Zhou Y., Anastopoulos I. (2021). Arbuscular Mycorrhizal Fungi-Induced Mitigation of Heavy Metal Phytotoxicity in Metal Contaminated Soils: A Critical Review. J. Hazard. Mater..

[30] Shi W., Zhang Y., Chen S., Polle A., Rennenberg H., Luo Z.-B. (2019). Physiological and Molecular Mechanisms of Heavy Metal Accumulation in Nonmycorrhizal versus Mycorrhizal Plants. Plant Cell Environ..

[31] Fan P., Wu L., Wang Q., Wang Y., Luo H., Song J., Yang M., Yao H., Chen S. (2023). Physiological and Molecular Mechanisms of Medicinal Plants in Response to Cadmium Stress: Current Status and Future Perspective. J. Hazard. Mater..

[32] Liang J., Wang Z., Ren Y., Jiang Z., Chen H., Hu W., Tang M. (2023). The Alleviation Mechanisms of Cadmium Toxicity in Broussonetia Papyrifera by Arbuscular Mycorrhizal Symbiosis Varied with Different Levels of Cadmium Stress. J. Hazard. Mater..

[33] Gil-Cardeza M.L., Ferri A., Cornejo P., Gomez E. (2014). Distribution of Chromium Species in a Cr-Polluted Soil: Presence of Cr(III) in Glomalin Related Protein Fraction. Sci. Total Environ..

[34] Chen X.W., Wu L., Luo N., Mo C.H., Wong M.H., Li H. (2019). Arbuscular Mycorrhizal Fungi and the Associated Bacterial Community Influence the Uptake of Cadmium in Rice. Geoderma.

[35] Cui G., Ai S., Chen K., Wang X. (2019). Arbuscular Mycorrhiza Augments Cadmium Tolerance in Soybean by Altering Accumulation and Partitioning of Nutrient Elements, and Related Gene Expression. Ecotoxicol. Environ. Saf..

[36] Rivera-Becerril F., van Tuinen D., Martin-Laurent F., Metwally A., Dietz K.-J., Gianinazzi S., Gianinazzi-Pearson V. (2005). Molecular Changes in *Pisum sativum* L. Roots during Arbuscular Mycorrhiza Buffering of Cadmium Stress. Mycorrhiza.

[37] Jia H., Wang X., Wei T., Zhou R., Muhammad H., Hua L., Ren X., Guo J., Ding Y. (2019). Accumulation and Fixation of Cd by Tomato Cell Wall Pectin under Cd Stress. Environ. Exp. Bot..

[38] Quinet M., Angosto T., Yuste-Lisbona F.J., Blanchard-Gros R., Bigot S., Martinez J.-P., Lutts S. (2019). Tomato Fruit Development and Metabolism. Front. Plant Sci..

[39] Wang Y., Jiang J., Zhao L., Zhou R., Yu W., Zhao T. (2018). Application of Whole Genome Resequencing in Mapping of a Tomato Yellow Leaf Curl Virus Resistance Gene. Sci. Rep..

[40] Shreevastav C., Subedi S., Gajurel S., Basnet P. (2022). A review on nutrient deficiency symptoms and effects on tomato plant. Food Agri Econ. Rev..

[41] Lafuente M.T., Sampedro R., Vélez D., Romero P. (2023). Deficient Copper Availability on Organoleptic and Nutritional Quality of Tomato Fruit. Plant Sci..

[42] Fei C., Zhang S., Sun Z., Ding X. (2024). Assessment of Magnesium Deficiency in Greenhouse Tomato Crops Grown on Calcareous Soil. Soil Use Manag..

[43] Hashimoto R., Ueta R., Abe C., Osakabe Y., Osakabe K. (2018). Efficient Multiplex Genome Editing Induces Precise, and Self-Ligated Type Mutations in Tomato Plants. Front. Plant Sci..

[44] Chen A., Hu J., Sun S., Xu G. (2007). Conservation and Divergence of Both Phosphate- and Mycorrhiza-Regulated Physiological Responses and Expression Patterns of Phosphate Transporters in *Solanaceous* Species. New Phytol..

[45] Liao D., Sun C., Liang H., Wang Y., Bian X., Dong C., Niu X., Yang M., Xu G., Chen A. (2022). SlSPX1-SlPHR Complexes Mediate the Suppression of Arbuscular Mycorrhizal Symbiosis by Phosphate Repletion in Tomato. Plant Cell.

[46] Bereczky Z., Wang H.-Y., Schubert V., Ganal M., Bauer P. (2003). Differential Regulation of Nramp and Irt Metal Transporter Genes in Wild Type and Iron Uptake Mutants of Tomato. J. Biol. Chem..

[47] Zhang H., Li G., Cao N., Yang H., Zhu F. (2021). Genome-Wide Identification and Expression Analysis of NRAMP Transporter Genes in *Cucumis sativus* and *Citrullus lanatus*. Can. J. Plant Sci..

[48] Ishida J.K., Caldas D.G.G., Oliveira L.R., Frederici G.C., Leite L.M.P., Mui T.S. (2018). Genome-Wide Characterization of the NRAMP Gene Family in *Phaseolus vulgaris* Provides Insights into Functional Implications during Common Bean Development. Genet. Mol. Biol..

[49] Li J., Duan Y., Han Z., Shang X., Zhang K., Zou Z., Ma Y., Li F., Fang W., Zhu X. (2021). Genome-Wide Identification and Expression Analysis of the NRAMP Family Genes in Tea Plant (*Camellia sinensis*). Plants.

[50] Mäser P., Thomine S., Schroeder J.I., Ward J.M., Hirschi K., Sze H., Talke I.N., Amtmann A., Maathuis F.J.M., Sanders D. (2001). Phylogenetic Relationships within Cation Transporter Families of Arabidopsis. Plant Physiol..

[51] Tang L., Mao B., Li Y., Lv Q., Zhang L., Chen C., He H., Wang W., Zeng X., Shao Y. (2017). Knockout of OsNramp5 Using the CRISPR/Cas9 System Produces Low Cd-Accumulating Indica Rice without Compromising Yield. Sci. Rep..

[52] Xiaohua H., Mo Y., Ji W., Yang X., Xie Z., Huang D., Li D., Tian L. (2022). The OsNramp4 Aluminum Transporter Is Involved in Cadmium Accumulation in Rice Grains. Reprod. Breed..

[53] Cailliatte R., Lapeyre B., Briat J.-F., Mari S., Curie C. (2009). The NRAMP6 Metal Transporter Contributes to Cadmium Toxicity. Biochem. J..

[54] Li J., Wang L., Zheng L., Wang Y., Chen X., Zhang W. (2018). A Functional Study Identifying Critical Residues Involving Metal Transport Activity and Selectivity in Natural Resistance-Associated Macrophage Protein 3 in Arabidopsis Thaliana. Int. J. Mol. Sci..

[55] Guo B., Liang Y., Fu Q., Ding N., Liu C., Lin Y., Li H., Li N. (2012). Cadmium Stabilization with Nursery Stocks through Transplantation: A New Approach to Phytoremediation. J. Hazard. Mater..

[56] Vision T.J., Brown D.G., Tanksley S.D. (2000). The Origins of Genomic Duplications in Arabidopsis. Science.

[57] Moore R.C., Purugganan M.D. (2005). The Evolutionary Dynamics of Plant Duplicate Genes. Curr. Opin. Plant Biol..

[58] Ullah I., Wang Y., Eide D.J., Dunwell J.M. (2018). Evolution, and Functional Analysis of Natural Resistance-Associated Macrophage Proteins (NRAMPs) from *Theobroma cacao* and Their Role in Cadmium Accumulation. Sci. Rep..

[59] Genome-Wide Identification and Expression Analysis of Metal Tolerance Protein (MTP) Gene Family in Soybean (Glycine Max) under Heavy Metal Stress | Molecular Biology Reports. https://link.springer.com/article/10.1007/s11033-022-08100-x.

[60] Chen Y., Zhao X., Li G., Kumar S., Sun Z., Li Y., Guo W., Yang J., Hou H. (2021). Genome-Wide Identification of the Nramp Gene Family in Spirodela Polyrhiza and Expression Analysis under Cadmium Stress. Int. J. Mol. Sci..

[61] Ehrnstorfer I.A., Geertsma E.R., Pardon E., Steyaert J., Dutzler R. (2014). Crystal Structure of a SLC11 (NRAMP) Transporter Reveals the Basis for Transition-Metal Ion Transport. Nat. Struct. Mol. Biol..

[62] Manatschal C., Pujol-Giménez J., Poirier M., Reymond J.-L., Hediger M.A., Dutzler R. (2019). Mechanistic Basis of the Inhibition of SLC11/NRAMP-Mediated Metal Ion Transport by Bis-Isothiourea Substituted Compounds. eLife.

[63] Ehrnstorfer I.A., Manatschal C., Arnold F.M., Laederach J., Dutzler R. (2017). Structural and Mechanistic Basis of Proton-Coupled Metal Ion Transport in the SLC11/NRAMP Family. Nat. Commun..

[64] Williams L.E., Pittman J.K., Hall J.L. (2000). Emerging Mechanisms for Heavy Metal Transport in Plants. Biochim. Biophys. Acta.

[65] Nakabayashi R., Yonekura-Sakakibara K., Urano K., Suzuki M., Yamada Y., Nishizawa T., Matsuda F., Kojima M., Sakakibara H., Shinozaki K. (2014). Enhancement of Oxidative and Drought Tolerance in Arabidopsis by Overaccumulation of Antioxidant Flavonoids. Plant J..

[66] Wang F., Kong W., Wong G., Fu L., Peng R., Li Z., Yao Q. (2016). AtMYB12 Regulates Flavonoids Accumulation and Abiotic Stress Tolerance in Transgenic Arabidopsis Thaliana. Mol. Genet. Genom..

[67] Pérez-Alonso M.-M., Sánchez-Parra B., Ortiz-García P., Santamaría M.E., Díaz I., Pollmann S. (2021). Jasmonic Acid-Dependent MYC Transcription Factors Bind to a Tandem G-Box Motif in the YUCCA8 and YUCCA9 Promoters to Regulate Biotic Stress Responses. Int. J. Mol. Sci..

[68] Zhou X., Yang Y. (2004). Differential Expression of Rice Nramp Genes in Response to Pathogen Infection, Defense Signal Molecules and Metal Ions. Physiol. Mol. Plant Pathol..

[69] Thomine S., Lelièvre F., Debarbieux E., Schroeder J.I., Barbier-Brygoo H. (2003). AtNRAMP3, a Multispecific Vacuolar Metal Transporter Involved in Plant Responses to Iron Deficiency. Plant J..

[70] Tang Z., Wang H.-Q., Chen J., Chang J.-D., Zhao F.-J. (2023). Molecular Mechanisms Underlying the Toxicity and Detoxification of Trace Metals and Metalloids in Plants. J. Integr. Plant Biol..

[71] Li Y., Ding L., Zhou M., Chen Z., Ding Y., Zhu C. (2023). Transcriptional Regulatory Network of Plant Cadmium Stress Response. Int. J. Mol. Sci..

[72] Peng F., Wang C., Zhu J., Zeng J., Kang H., Fan X., Sha L., Zhang H., Zhou Y., Wang Y. (2018). Expression of TpNRAMP5, a Metal Transporter from Polish Wheat (*Triticum Polonicum* L.), Enhances the Accumulation of Cd, Co and Mn in Transgenic Arabidopsis Plants. Planta.

[73] Chang J.-D., Huang S., Yamaji N., Zhang W., Ma J.F., Zhao F.-J. (2020). OsNRAMP1 Transporter Contributes to Cadmium and Manganese Uptake in Rice. Plant Cell Environ..

[74] Li H., Chen X.W., Wu L., Luo N., Huang W.X., Mo C.H., Wong M.H. (2020). Effects of Arbuscular Mycorrhizal Fungi on Redox Homeostasis of Rice under Cd Stress. Plant Soil.

[75] Pan J., Cao S., Xu G., Rehman M., Li X., Luo D., Wang C., Fang W., Xiao H., Liao C. (2023). Comprehensive Analysis Reveals the Underlying Mechanism of Arbuscular Mycorrhizal Fungi in Kenaf Cadmium Stress Alleviation. Chemosphere.

[76] Liu D., Zheng K., Wang Y., Zhang Y., Lao R., Qin Z., Li T., Zhao Z. (2022). Harnessing an Arbuscular Mycorrhizal Fungus to Improve the Adaptability of a Facultative Metallophytic Poplar (*Populus yunnanensis*) to Cadmium Stress: Physiological and Molecular Responses. J. Hazard. Mater..

[77] Liu H., Yuan M., Tan S., Yang X., Lan Z., Jiang Q., Ye Z., Jing Y. (2015). Enhancement of Arbuscular Mycorrhizal Fungus (*Glomus versiforme*) on the Growth and Cd Uptake by Cd-Hyperaccumulator *Solanum nigrum*. Appl. Soil. Ecol..

[78] Liu M., Sun J., Li Y., Xiao Y. (2017). Nitrogen Fertilizer Enhances Growth and Nutrient Uptake of *Medicago sativa* Inoculated with *Glomus tortuosum* Grown in Cd-Contaminated Acidic Soil. Chemosphere.

[79] Ouziad F., Hildebrandt U., Schmelzer E., Bothe H. (2005). Differential Gene Expressions in Arbuscular Mycorrhizal-Colonized Tomato Grown under Heavy Metal Stress. J. Plant Physiol..

[80] Kabir A.H., Debnath T., Das U., Prity S.A., Haque A., Rahman M.M., Parvez M.S. (2020). Arbuscular Mycorrhizal Fungi Alleviate Fe-Deficiency Symptoms in Sunflower by Increasing Iron Uptake and Its Availability along with Antioxidant Defense. Plant Physiol. Biochem..

[81] Watts-Williams S.J., Tyerman S.D., Cavagnaro T.R. (2017). The Dual Benefit of Arbuscular Mycorrhizal Fungi under Soil Zinc Deficiency and Toxicity: Linking Plant Physiology and Gene Expression. Plant Soil..

[82] Cardini A., Pellegrino E., Declerck S., Calonne-Salmon M., Mazzolai B., Ercoli L. (2021). Direct Transfer of Zinc between Plants Is Channelled by Common Mycorrhizal Network of Arbuscular Mycorrhizal Fungi and Evidenced by Changes in Expression of Zinc Transporter Genes in Fungus and Plant. Environ. Microbiol..

[83] Liu J., Liu J., Chen A., Ji M., Chen J., Yang X., Gu M., Qu H., Xu G. (2016). Analysis of Tomato Plasma Membrane H^+^-ATPase Gene Family Suggests a Mycorrhiza-Mediated Regulatory Mechanism Conserved in Diverse Plant Species. Mycorrhiza.

[84] Studer G., Tauriello G., Bienert S., Waterhouse A.M., Bertoni M., Bordoli L., Schwede T., Lepore R. (2019). Modeling of Protein Tertiary and Quaternary Structures Based on Evolutionary Information. Methods Mol. Biol..

[85] Biasini M., Bienert S., Waterhouse A., Arnold K., Studer G., Schmidt T., Kiefer F., Gallo Cassarino T., Bertoni M., Bordoli L. (2014). SWISS-MODEL: Modelling Protein Tertiary and Quaternary Structure Using Evolutionary Information. Nucleic Acids Res..

[86] Bertoni M., Kiefer F., Biasini M., Bordoli L., Schwede T. (2017). Modeling Protein Quaternary Structure of Homo- and Hetero-Oligomers beyond Binary Interactions by Homology. Sci. Rep..

[87] Chen C., Chen H., Zhang Y., Thomas H.R., Frank M.H., He Y., Xia R. (2020). TBtools: An Integrative Toolkit Developed for Interactive Analyses of Big Biological Data. Mol. Plant.

