# Supplementary material for: Genome-Wide Identification and Expression Analysis of SlNRAMP Genes in Tomato under Nutrient Deficiency and Cadmium Stress during Arbuscular Mycorrhizal Symbiosis
Source: Int J Mol Sci. 2024 Jul 29;25(15):8269. doi: 10.3390/ijms25158269 (PMC11311520; doi:10.3390/ijms25158269)
Supplement: Supplementary file 1 [file ijms-25-08269-s001.zip › ijms-3129480-supplementary.pdf]

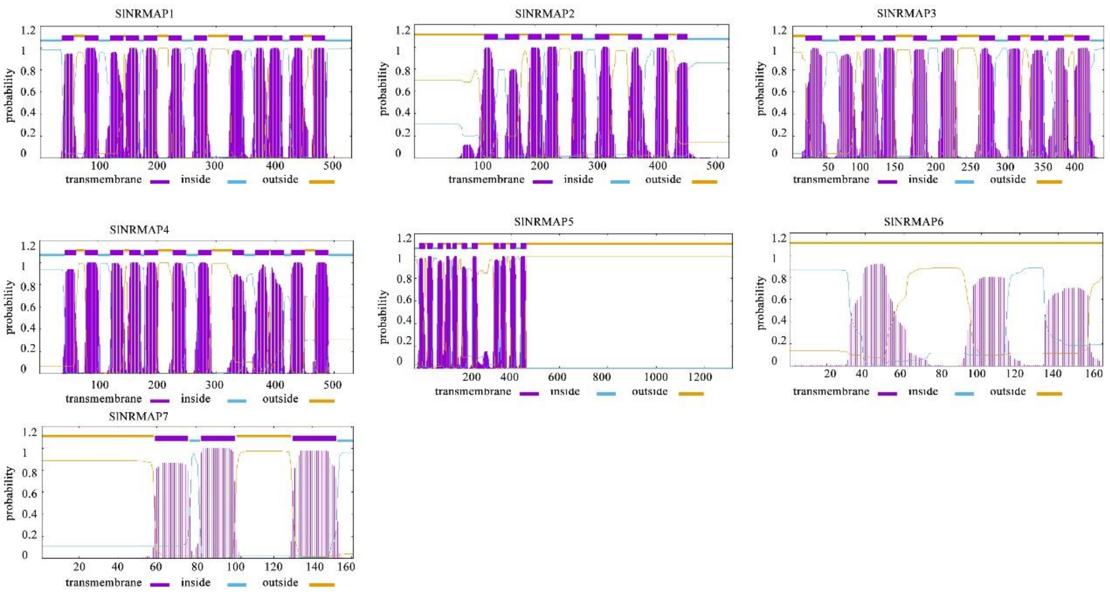

Figure S1. Prediction of transmembrane structure of the SlNRAMP protein family.

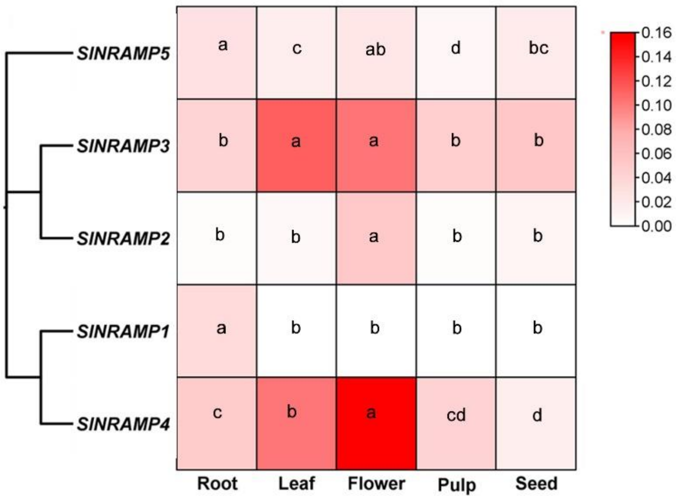

Figure S2. Tissue-specific expression profiles of the SlNRAMP genes was performed in various tomato tissues.

The 14-day-old seedlings were transplanted and grown in greenhouse for four months, then SlNRMAP gene family expression levels were determined in different tissues. leaves, roots, flowers, pulps and seeds were separately harvested for qRT-PCR analysis. Fold-changes of genes expression were normalized against the reference gene *SlActin* using $2^{\wedge(-\Delta Ct)}$ values. Data (means ± SE, n = 4) sharing the same letter(s) above the error bars are not significantly different at the 0.05 level based on the Duncan's test.

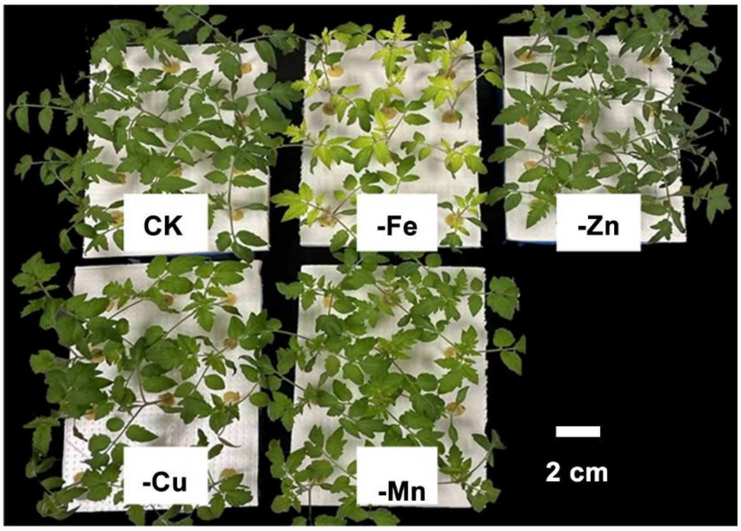

Figure S3. Photograph of the treatments

The 14-day-old seedlings were subjected to four nutrient deficiencies (-Fe, -Cu, -Zn, -Mn) for 7 days, and then the shoots and roots elements concentration, as well as SlNRMAP gene family expression levels were determined.

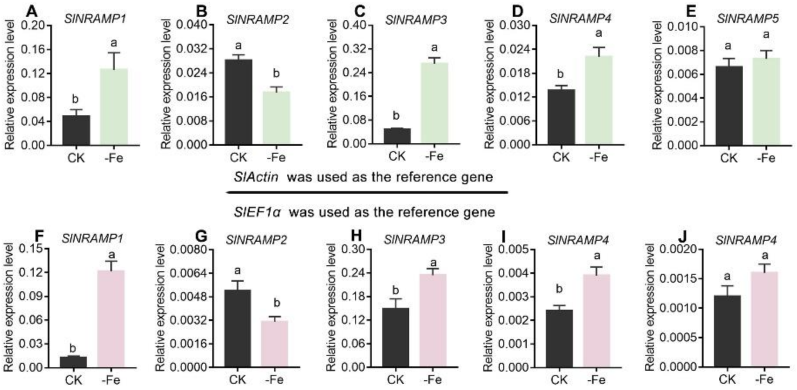

Figure S4 Expression levels of the *SlNRAMP1* gene under Fe deficiency using two reference genes

The 14-day-old seedlings were subjected to four nutrient deficiencies (-Fe, -Cu, -Zn, -Mn) for 7 days, and then *SlNRMAP1* expression levels in roots was determined. The gene expression level was normalized against the reference gene *SlActin* (A-E) and *SlEF1α*(F-J) using $2^{\wedge(-\Delta Ct)}$ values. Data (means ±SE, n = 5), different letters indicate significant differences (p <0.05, Duncan's test)

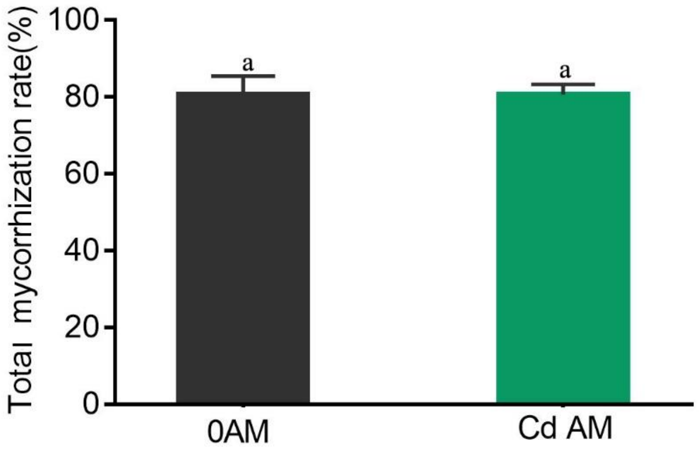

Figure S5 Total mycorrhization of mycorrhizal tomato plants grown in Cd free (0AM) and 100 μM Cd (CdAM) for 35 days.

The 14-day-old seedlings were inoculated with or without AMF under Cd stress for 35 days, and then total mycorrhization of mycorrhizal tomato plants was determined. Data (means ±SE, n = 3), Different letters indicate significant differences ($p < 0.05$, Duncan's test)

Table S1 The list of SlNRAMP family gene in different plants

plant species	Gene name	Geng ID	plant species	Gene name	Geng ID
	AtNRAMP1	AT1G80830.1		*GmNRAMP1a*	Glyma.01G190700.1
	AtNRAMP2	AT1G47240.1		*GmNRAMP1b*	Glyma.11G051500.1
	AtNRAMP3	AT2G23150.1		*GmNRAMP2a*	Glyma.05G101700.1
	AtNRAMP4	AT5G67330.1		*GmNRAMP2b*	Glyma.17G165200.1
	AtNRAMP5	AT4G18790.1		*GmNRAMP3a*	Glyma.04G044000.1
Arabidopsis thaliana	*AtNRAMP6*	AT1G15960.1		*GmNRAMP3b*	Glyma.06G044200.1
	StNRAMP1	PGSC0003DMG400023310		*GmNRAMP4b*	Glyma.16G027800.1
	StNRAMP2	PGSC0003DMT400064287		*GmNRAMP5a*	Glyma.07G023600.2
	StNRAMP3	PGSC0003DMT400055473		*GmNRAMP5b*	Glyma.08G218200.1
	StNRAMP4	PGSC0003DMT400055484		*GmNRAMP6a*	Glyma.13G369900.1
Solanum tuberosum	*StNRAMP5*	PGSC0003DMT400023921	*Glycine Max*	*GmNRAMP6b*	Glyma.15G003500.1
	OsNRAMP1	Os07t0258400-03		*GmNRAMP7*	Glyma.06G115800.2
	OsNRAMP2	Os03t0208500-01		*SlNRMAP1*	Solyc11g018530.2.1
	OsNRAMP3	Os06t0676000-01		*SlNRMAP2*	Solyc04g078250.3.1
	OsNRAMP4	Os02t0131800-01		*SlNRMAP3*	Solyc02g092800.3.1
	OsNRAMP5	Os07t0257200-01		*SlNRMAP4*	Solyc03g116900.3.1
	OsNRAMP6	Os01t0503400-03	*Solanum Lycopersicum*	*SlNRMAP5*	Solyc09g007870.3.1
Oryza sativa	*OsNRAMP7*	Os12t0581600-01			

Table S2 Analysis of the ten conserved motifs of SlNRAMP family proteins in tomato

Motif	Logo	Sequence	Width	Annotation
1	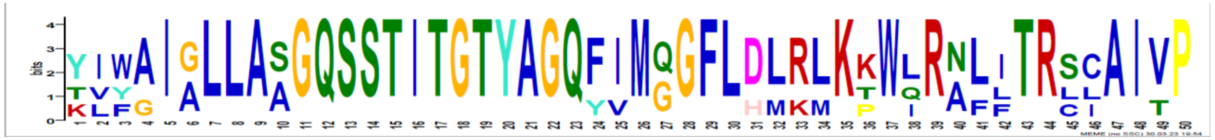	YIWAIGLLAAGQSSTITGTYAGQFIMGGFLDLRLKKWLRALITRSCAIVP	50	NRAMP
2	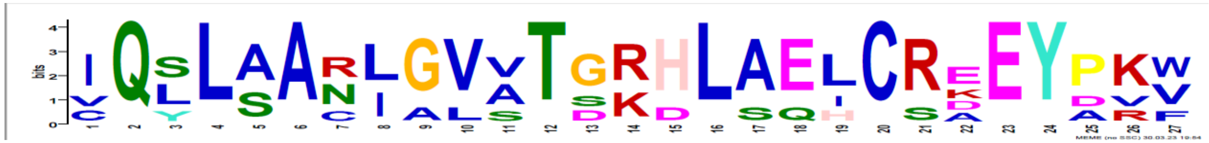	IQLLAARJGVATGRHLAELCREEYPKW	27	NRAMP
3	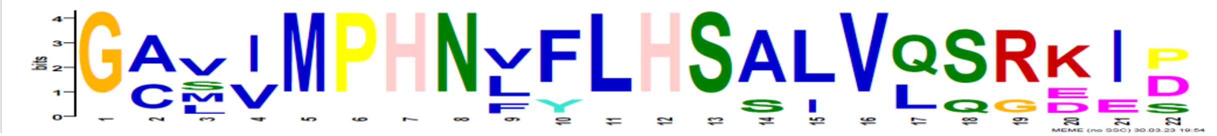	GAVIMPHNLFLHSALVQSRKIP	22	NRAMP
4	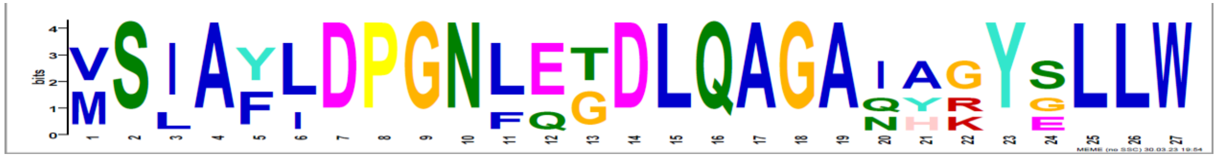	VSIAYLDPGNLEGDLQAGAIAGYSLLW	27	NRAMP
5	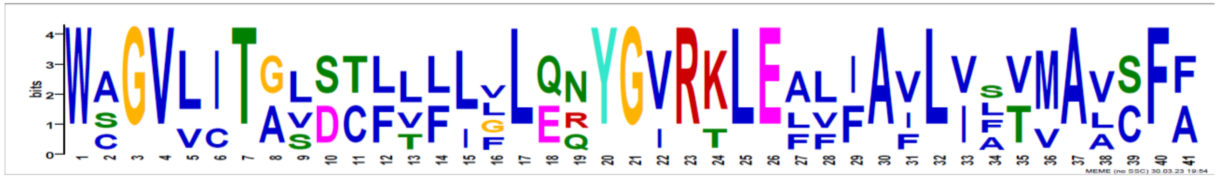	WAGVLITGLDCFLFLLLZNYGVRKLEALFAVLIAVMAVSF	41	NRAMP

6	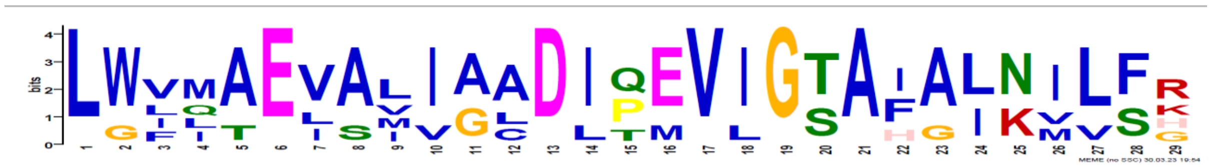	LWVMAEVAJIAADIPEVIGTAFAJNILFR	29	NRAMP
7	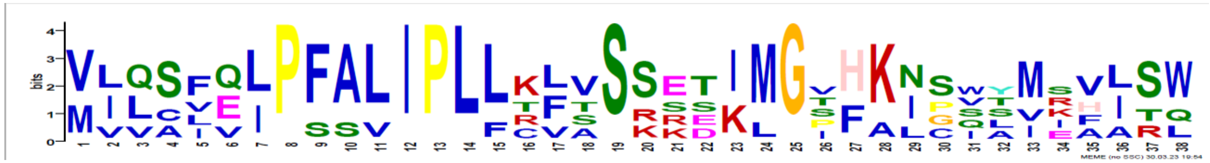	VILSFZJPFALIPLLKLVSSETIMGVHKISVSMKVJSW	38	NRAMP
8	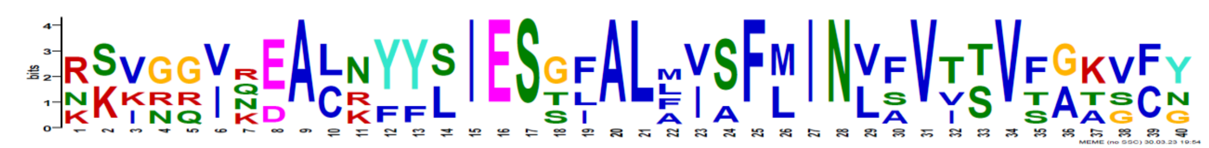	RKVGGIKEALNYYLIESGFALLISFLINLFVTSVFGKVFY	40	NRAMP
9	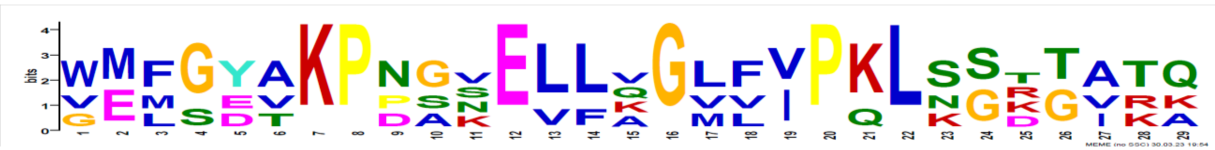	WEFGYAKPNGNELLAGLFIPKLSGKGATQ	29	NRAMP
10	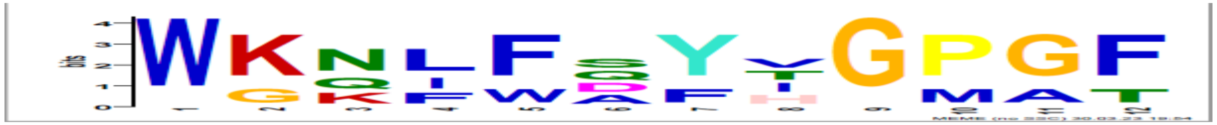	WKNLFAYVGPGF	12	NRAMP

Table S3 The genomic location of SlNRAMP family genes

Gene name	Gene ID	Genomic Location
SlNRAMP1	Solyc11g018530.2.1	Chr11:8645242-8648856
SlNRAMP2	Solyc04g078250.3.1	Chr4:63109871-63113592
SlNRAMP3	Solyc02g092800.3.1	Chr2:54402272-54405963
SlNRAMP4	Solyc03g116900.3.1	Chr3:67650010-67659122
SlNRAMP5	Solyc09g007870.3.1	Chr9:1399607-1407814

Table S4 The analysis of collinear gene pairs between different plants

Collinear plant 1	Collinear plant 2	collinear gene pairs
Arabidopsis thaliana	*Glycine Max*	*AtNRAMP2*： *GmNRAMP3a/GmNRAMP3b*; *AtNRAMP6*： *GmNRAMP5a/GmNRAMP5b/GmNRAMP6a/ GmNRAMP6b*; *AtNRAMP3*： *GmNRAMP1a/GmNRAMP2a/GmNRAMP3b/ GmNRAMP1b/GmNRAMP2b*; *AtNRAMP4*: *GmNRAMP1a/ GmNRAMP3a/GmNRAMP2a/GmNRAMP3b/GmNRAMP1b/ GmNRAMP2b*
Glycine Max	*Solanum Lycopersicon*	*SlNRAMP5:* Glyma.03G181400.3/Glyma.10G058300.4/Glyma.13G145100.4/Glyma.19G182151.1; Solyc04g078220.3.1*:* *GmNRAMP1a/GmNRAMP3a/GmNRAMP2a/GmNRAMP3b/GmNRAMP1b/GmNRAMP2b*; *SlNRAMP4*： *GmNRAMP5a/ GmNRAMP5b/GmNRAMP6a/GmNRAMP6b/*; *SlNRAMP3: GmNRAMP1a/GmNRAMP3a/GmNRAMP2a/GmNRAMP3b/GmNRAMP1b/GmNRAMP2b*
Solanum lycopersicum	*Solanum tuberosum*	*SlNRAMP5:StNRAMP4*<br>*SlNRAMP3:StNRAMP2/StNRAMP3*<br>*SlNRAMP1: StNRAMP5*
Solanum tuberosum	*Medicago sativa*	*MtNRAM5: StNRAM2/StNRAM3*<br>*MtNRAM1: StNRAM5*<br>*MtNRAM4: StNRAM2/StNRAM3*<br>*MtNRAM7: StNRAM2/StNRAM3*
Medicago sativa	*Oryza sativa*	*OsNRAMP2: MtNRAM4*<br>*OsNRAMP2: MtNRAM7*<br>*OsNRAMP2: MtNRAM5*

Table S5 The best templates of SlNRAMP family proteins selected from the SwissModel template library for building 3D structure models

Gene name	Template	Sequence identity (%)	Range	Coverage	GMQE	QMEANDisCo global score	Description
SlNRAMP1	5m8k.1.A	33.04	29 - 492	0.86	0.59	0.64 ± 0.05	Divalent metal cation transporter MntH
SlNRAMP2	5m8k.1.A	31.3	72 - 487	0.79	0.53	0.63 ± 0.05	Divalent metal cation transporter MntH
SlNRAMP3	5m8k.1.A	31.7	1 - 433	0.96	0.66	0.64 ± 0.05	Divalent metal cation transporter MntH
SlNRAMP4	5m8k.1.A	34.37	37 - 496	0.85	0.58	0.65 ± 0.05	Divalent metal cation transporter MntH
SlNRAMP5	5m8k.1.A	24.56	11 - 465	0.34	0.20	0.59 ± 0.05	Divalent metal cation transporter MntH

Table S6 The cis-acting elements in the promoter regions of SlNRAMP family genes

Classification	Motif name	Function	Number of motif of *SlNRMAP* family genes				
			SlNRAM P1	*SlNRAM P2*	*SlNRAM P3*	*SlNRAM P4*	*SlNRAM P5*
Gene transcription	CAAT-box	common cis-acting element in promoter and enhancer regions	16	12	15	16	17
	TATA-box	core promoter element around -30 of transcription start	110	38	50	91	61
Plant growth and development	chs-CMA1a	part of a light responsive element			1		
	LAMP-element	part of a light responsive element	1	1			
	TCT-motif	part of a light responsive element	3		1		
	GATA-motif	part of a light responsive element	1			1	
	AE-box	part of a module for light response	3	2			
	I-box	part of a light responsive element	1				1
	AT1-motif	part of a light responsive module		1			
	Gap-box	part of a light responsive element					1
	TCCC-motif	part of a light responsive element		1			
	ATCT-motif	part of a conserved DNA module involved in light responsiveness			2	3	
	Box 4	part of a conserved DNA module involved in light responsiveness	2	1	4	2	
	ACE	cis-acting element involved in light responsiveness		1			
	G-Box	cis-acting regulatory element involved in light responsiveness		3	3	1	1
	3-AF1 binding site	light responsive element					1

	GT1-motif	light responsive element	1	1	1		
	CAT-box	cis-acting regulatory element related to meristem expression				2	
	O2-site	cis-acting regulatory element involved in zein metabolism regulation	2	1			1
	circadian	cis-acting regulatory element involved in circadian control					2
	ARE	cis-acting regulatory element essential for the anaerobic induction					3
	TC-rich repeats	cis-acting element involved in defense and stress responsiveness	1			1	2
	W box	wound responsive		3		1	
	STRE	heat shock protein-related element	1	2	1	1	3
	WUN-motif	Wound-responsive element	1	2			
Abiotic and biotic stresses	MBS	MYB binding site involved in drought-inducibility	2	1		3	1
	CCAAT-box	MYBHv1 binding site				1	
	MYB	MYB binding site	4	4	3	9	10
	MYB recognition site	MYB recognition site				1	
	Myb	MYb binding site		1	1	4	1
	MYB-like sequence	MYB-like sequence	1		1		2
	MYC	MYC binding site	2	1	3	4	4
	MRE	MYB binding site involved in light responsiveness	1		3		
Phytohormone reponsive	ABRE3a	cis-acting element involved in the abscisic acid responsiveness		1		1	

ABRE4	cis-acting element involved in the abscisic acid responsiveness		1		1	
as-1	SA and oxidative responsiveness	1	1		3	1
TCA	cis-acting element involved in salicylic acid responsiveness		1	2		
ERE	ethylene response element	1	3	6	4	
TCA-element	cis-acting element involved in salicylic acid responsiveness				2	2
ABRE	cis-acting element involved in the abscisic acid responsiveness		2	3	1	
TGA-element	auxin-responsive element		1	1		
AuxRR-core	cis-acting regulatory element involved in auxin responsiveness				1	
GARE-motif	gibberellin-responsive element		1		1	1
TGACG-motif	cis-acting regulatory element involved in the MeJA-responsiveness	1	1		3	1
CGTCA-motif	cis-acting regulatory element involved in the MeJA-responsiveness	1	1		3	1

Table S7 Expression levels of SlNRAMP family genes in tomato were subjected to Fe, Cu, Mn, Zn deficiency.

Treatments		*SlNRAMP1*	*SlNRAMP2*	*SlNRAMP3*	*SlNRAMP4*	*SlNRAMP5*
	CK	0.048±0.012b	0.028±0.002b	0.049±0.003b	0.014±0.001c	0.007±0.001a
	-Fe	0.126±0.028a	0.017±0.002c	0.270±0.020a	0.022±0.002b	0.006±0.000a
Roots	-Zn	0.037±0.005b	0.028±0.003b	0.054±0.002b	0.015±0.001c	0.006±0.000a
	-Cu	0.013±0.001b	0.039±0.004a	0.035±0.001b	0.029±0.002a	0.005±0.000a
	-Mn	0.069±0.028ab	0.029±0.006b	0.046±0.003b	0.026±0.002ab	0.007±0.000a
	CK	0.0001±0.00005b	0.017±0.002a	0.074±0.004b	0.036±0.005b	0.019±0.003b
	-Fe	0.0004±0.00012a	0.006±0.001b	0.365±0.021a	0.103±0.022a	0.027±0.002a
Leaves	-Zn	0.0002±0.0005ab	0.017±0.001a	0.055±0.004b	0.031±0.002b	0.025±0.002a
	-Cu	0.0001±0.004b	0.018±0.002a	0.081±0.003b	0.036±0.006b	0.016±0.001b
	-Mn	0.0002±0.0006ab	0.014±0.001a	0.057±0.005b	0.026±0.003b	0.024±0.001a

The 14-day-old seedlings were subjected to four nutrient deficiencies (-Fe, -Cu, -Zn, -Mn) for 7 days, and then SlNRMAP gene family expression levels in roots and leaves was determined. The gene expression level was normalized against the reference gene *SlActin* using $2^{\wedge(-\Delta Ct)}$ values. Data (means ±SE, n = 5), Different letters indicate significant differences ($p < 0.05$, Duncan's test)

Table S8 Primers of SlNRAMP family genes selected for qRT-PCR analysis

Gene	NCBI Acc. Num.	Forward (5' to 3')	Reverse (5' to 3')
SlActin	112697914	TTCCGTTGCCCAGAGGTCCT	TCGCCCTTTGAAATCCACATC
SlNRMAP1	112706038	AGTTGGGACCACACAAGAAC	TGTTGCTTATCAGCCAACC
SlNRMAP2	112717764	ACTATGCTGGTCAATGAGACA	AACGCTGGCAGTTTCTCAA
SlNRMAP3	112766867	TGGTTAACTGGATTGTTGGCTGCTGGAC	ATGAATTCAAAGAAGGGGGATCAGGGCA
SlNRMAP4	112706457	ACGTATGCAGTTGCCTCAG	GACGATACGAGTTCTAACTG
SlNRMAP5	112764447	CAGAGTGTAATCAACTTGGTGC	GGGTTACCAAGGATTTTTCCAC
SlEF1α	X14449	GGAACTTGAGAAGGAGCCTAAG	CAACACCAACAGCAACAGTCT
